# Real‐Time 3D Ultrasound Imaging with an Ultra‐Sparse, Low Power Architecture

**DOI:** 10.1002/adhm.202505310

**Published:** 2026-01-29

**Authors:** Colin Marcus, Md Osman Goni Nayeem, Aastha Shah, Jason Hou, Shrihari Viswanath, Maya Eusebio, David Sadat, Anantha P. Chandrakasan, Tolga Ozmen, Canan Dagdeviren

**Affiliations:** ^1^ Media Lab Massachusetts Institute of Technology Cambridge Massachusetts USA; ^2^ Department of Electrical Engineering and Computer Science Massachusetts Institute of Technology Cambridge Massachusetts USA; ^3^ Division of Surgical Oncology Massachusetts General Hospital Harvard Medical School Boston Massachusetts USA

**Keywords:** breast imaging, chirp data acquisition system, real‐time 3D imaging, sparse array, ultrasound imaging

## Abstract

Effective resource‐constrained volumetric ultrasound imaging requires compact, low‐power systems capable of wide‐angle real‐time 3D imaging to accommodate small changes in placement by the operator. However, obtaining such images requires an excessive O(N^2^) channel count, bulky electronics, and high power consumption. We introduce an end‐to‐end system architecture to enable high‐resolution, real‐time 3D ultrasound imaging in a portable form factor. We present: a convolutional optimally distributed array (CODA) geometry that drastically reduces the number of elements (from 1024 to 128), a novel chirped data acquisition (cDAQ) architecture that enhances imaging depth while operating with a 25.3 dB lower transmit amplitude than a pulsed system, and an associated new signal processing methodology. We experimentally demonstrate our system's ability to perform deep (> 11 cm), high axial resolution (< 600 µm), and wide‐angle (57°) imaging, while simultaneously reducing power consumption (29.6x reduction) and drive voltage (18 V). We validated our system in vitro and further performed in vivo human trials, demonstrating the ability to detect both tumors and cysts in breast tissue. This new architectural approach will unlock a new class of medical devices with enhanced diagnostic and long‐term monitoring capabilities and open up future wearable designs of real‐time 3D ultrasound systems.

## Graphical abstract

This article presents a novel, ultra‐sparse ultrasound architecture that paves the way for wearable real‐time 3D imaging. By integrating a unique convolutional array with chirped data acquisition, the system achieves high‐resolution volumetric scans at a fraction of the power and hardware complexity. Discover how this portable innovation unlocks accessibility and new possibilities for wearable medical diagnostics and long‐term monitoring.

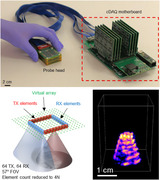

## 1 Introduction

Ultrasonography is in widespread use as a safer and less expensive imaging modality compared to X‐ray, computed tomography, and magnetic resonance imaging [1, 2, 3, 4, 5]. The developing field of ultrasound imaging has attracted considerable interest for its potential to enable ambulatory, at home, and long‐term biomedical applications [6, 7]. Recent advancements include imaging of cerebral blood flow [8], cardiac monitoring [9], portable/wearable electronics [10, 11], bioadhesive ultrasound couplants [12], measurement of bladder volume [13], and long‐term monitoring for breast cancer detection, especially in young women with dense breast composition [14]. While these works have introduced methods of fabricating conformable arrays, explored novel acoustic coupling materials, and have demonstrated the potential for ultrasound to provide a diagnostic alternative to X‐ray mammography — transducer misalignment remains a fundamental challenge, especially for patch‐type devices in attachable/wearable form factor. Unlike in traditional ultrasound, where an operator can move the probe and search for a target, wearable ultrasound transducers are meant to sit stationary on the body. Most ultrasound probes restrict imaging to a narrow 2D plane (typically <3 mm thick), which may not intersect the region of interest without precise manual positioning. Moreover, clinical imaging systems are inherently designed for real‐time 2D acquisition. Even with careful placement, imperceptible tilting of the transducer can cause a large displacement of the imaging plane. Proposed solutions have included adding additional arrays or overlaying multiple 1D arrays to take multiple B‐mode slices at different angles, however these are insufficient to address the problem [9, 13]. Attempting to intersect a randomly located target within a volume using thin planar slices is simply not reliable enough to form the basis of an operator‐independent medical technology.

The direct solution would be wide‐angle volumetric imaging, however existing 3D video imaging architectures present critical tradeoffs. Currently, three main classes of architectures exist: full matrix, row‐column, and sparse arrays. Full matrix arrays implement a full 2D matrix of elements, offering the best performance and a wide field of view (FOV), but also leading to very high element counts (O(N^2^) scaling with current state‐of‐the‐art commercial systems supporting up to 256 × 256 or 65 536 elements (ClearVue, Philips)). Such arrays are challenging and expensive to fabricate (especially interconnect wiring), and require bulky and power‐intensive electronics to interface with all the elements. While at least one full matrix array has been presented in the wearable format, the element count (16 × 16 or 256) was insufficient to produce high resolution images [8]. Additionally, the O(N^2^) scaling is a key blocker for achieving high resolution real‐time imaging in portable/wearable devices, and scaling up to a 32 × 32 device would require 4 Verasonics Vantage 256 systems for data acquisition, each weighing approximately 90 lbs. Although time‐multiplexed architectures could in principle reduce electronic complexity and hardware demands, they do so at the cost of temporal resolution, limiting real‐time volumetric imaging performance.

Row‐column addressed arrays reduce the wiring complexity by operating the transducer as a 1D linear array on one axis for transmit (TX), and switching to the orthogonal axis for receive (RX) [15]. This reduces the number of signals to O(4N). However, row‐column arrays can only form images in the small region where the orthogonal TX and RX imaging planes overlap. Thus, the imaging region is confined to the footprint of the array itself, forming a narrow “pencil beam” that actually poses even harsher requirements for precise positioning and tilting than the B‐mode images. Some recent work has attempted to expand the FOV by placing an acoustic lens in front of the row‐column array, but diffractive and attenuative effects of the lens resulted in degraded image resolution and contrast [16].

Finally, sparse arrays are essentially full matrix arrays with most of the elements removed. As such, they are capable of wide‐angle imaging. A number of designs have been proposed over the years with random, optimized‐random, and other geometries [17, 18]. The common theme is that as the number of elements is reduced from O(N^2^) toward O(4N), the arrays increasingly suffer from image artifacts, elevated sidelobes, poor contrast performance, and low imaging depth due to the reduced active area of the array. Existing techniques, such as increasing TX drive voltage, coded excitations [19, 20], or waveform averaging, can compensate for the reduced imaging depth, but compromise safety, increase power consumption, and cause excessive heating.

Thus, there are three key challenges that must be addressed to enable real‐time 3D ultrasound imaging in a portable form factor for broader accessibility. First, a sparse array geometry is needed that can provide a high image resolution and a wide FOV, while using a minimal number of channels. Second, an electronic and signal processing approach is required to compensate for the low signal‐to‐noise ratio (SNR) of such an array, while using low voltages and power consumption. Third, a frequency compression technique is needed that can further reduce the data acquisition and processing burden. Although convolutional beamforming has been reported [17], no existing system integrates it into a low‐power architecture with on‐device down conversion to achieve real‐time 3D imaging.

In this work, we introduce a new, end‐to‐end ultrasound architecture including a box‐shaped array with separated TX and RX subarrays, co‐developed alongside an electronics analog front end which uses continuous wave transmits and low frequency receiver circuits to greatly boost the SNR while simultaneously consuming less power than standard ultrasound systems. We use chirp excitations to compress frequency information, reducing ADC and data transmission demands, which enables low‐latency real‐time data transfer to the GPU for live image reconstruction. We validate the system performance for both B‐mode and real‐time 3D imaging, demonstrating comparable imaging depth while operating with a 25.3 dB lower transmit amplitude than a pulsed system, axial resolution under 600 µm, and low ADC power consumption of 1.50 mW/(channel·MHz). Further, we demonstrate the cDAQ's capability to detect both tumors and cysts in human breast tissue by conducting imaging on both in vitro phantoms and in vivo human subjects.

## 2 Results

### 2.1 Convolutional Optimally Distributed Array Design

**FIGURE 1:**
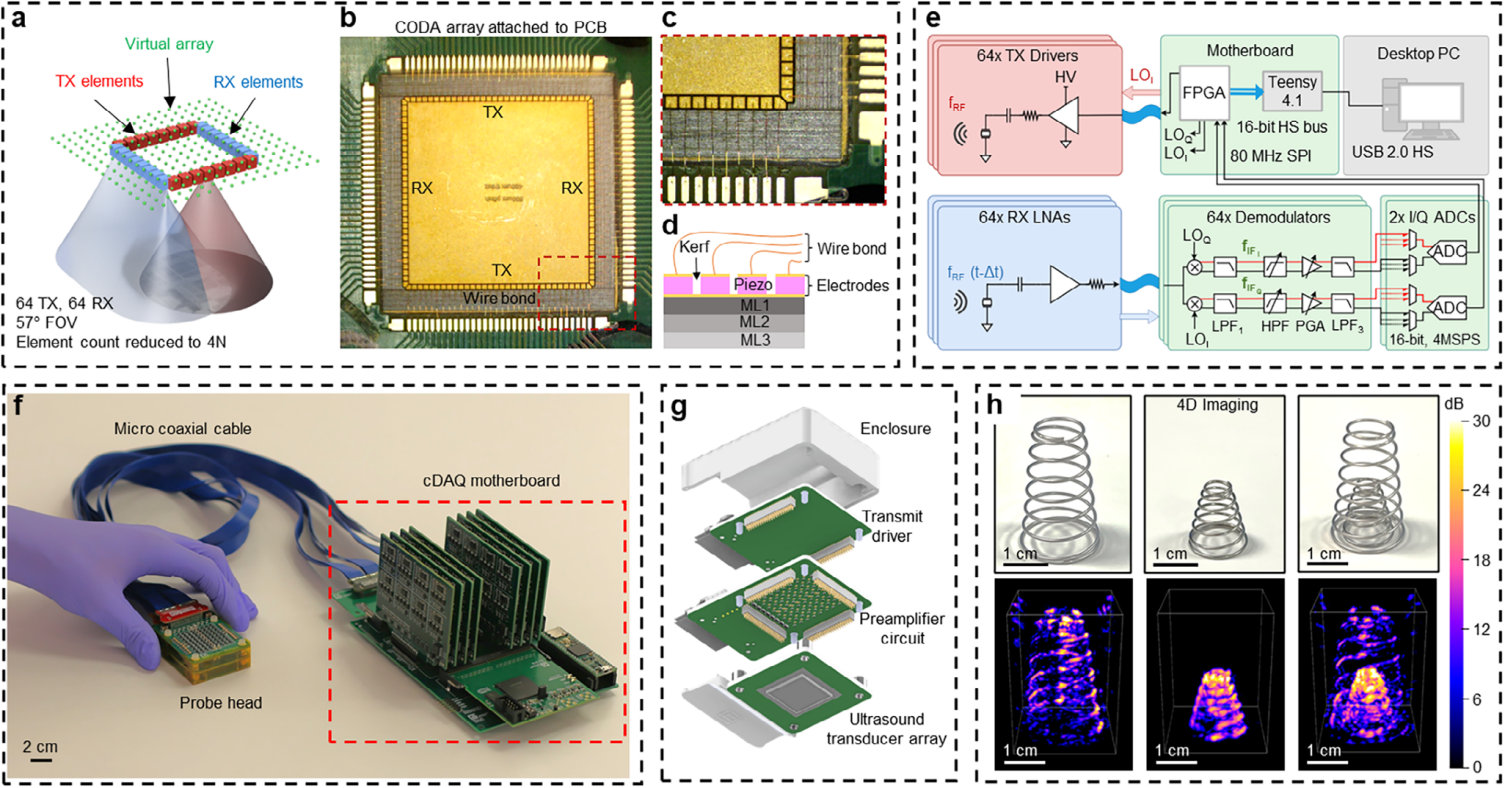
Real‐time 3D ultrasound imaging system architecture. (a) Conceptual diagram illustrating the CODA array design, with dedicated transmit (TX) and receive (RX) elements and the corresponding uniformly sampled “virtual array.” (b) Actual CODA array with 64 TX and 64 RX elements, mounted in a carrier PCB. (c) Magnified view of the CODA array showing the element geometry and wire bonding. (d) Schematic side view of the CODA array showing the three matching layers, solid bottom ground electrode, and machined piezoelectric ceramic and top electrodes. (e) Block diagram of the cDAQ showing the transmit driver (red) and receive LNA (blue), connected via micro coaxial cables to the motherboard (green). The motherboard contains modules performing downconversion, filtering, amplification, and sampling of the received signals. An FPGA generates excitation signals, receives data from the ADCs, and communicates with a PC via a Teensy 4.1 module. (f) Photograph of the cDAQ system comprising the probe head connected by a micro coaxial cable to the motherboard. (g) Exploded view of the probe head, including the CODA array, LNA circuit, transmit driver circuit, and 3D printed enclosure. (h) Photographs and corresponding volumetric ultrasound images of conical springs acquired by the CODA array and cDAQ system (chirp duration: 8.7 ms, bandwidth: 1–5 MHz, acquisition depth: 10 cm).

To address the requirement for a highly sparse array with deep and wide‐angle imaging, we developed a box‐shaped array (Figure 1a) with separate and orthogonal TX and RX subarrays. The convolution of these TX and RX subarrays defines an effective acoustic aperture or “virtual array,” the size and distribution of which determines the imaging resolution [18]. Our convolutional optimally distributed array (CODA) has an acoustic aperture with no repeated samples and no gaps, meaning it achieves the sharpest far‐field image resolution with minimal artifacting behavior. The small elements have a wide angle of sensitivity, allowing imaging well outside the array footprint. As a result, our CODA array with O(4N) elements achieves the same combined wide‐angle imaging and far‐field resolution as a full matrix array with O(N^2^) elements. It should be noted that the box geometry is not a unique solution to the CODA problem. For instance, an “X” shaped array would convolve to the same unity‐valued and gapless virtual array. The advantage of our box‐shaped array is that among all possible CODA arrays, it consumes the smallest physical footprint for a given virtual array size (Figure S1). One important caveat with the CODA array is that like other sparse arrays, sensitivity and contrast degrade in the extreme near field, in this work within approximately 1.0 cm of the array (Figure S2). It should be noted that another box‐shaped array has been mentioned in the theoretical literature [18], but with all elements wired as transceivers (TX and RX). As a result, it had double the electronics requirements of our CODA array, while not providing any increased resolution and having a non‐optimal and highly distorted spatial apodization.

The CODA array produced in this work (Figure 1b) features 128 elements and matches the aperture size of a 1024‐element full‐×matrix array, achieving an 8x reduction in total elements and a 16x reduction in RX elements (Figure S1). The unusual element geometries and half‐pitch spacing between the subarrays precluded the use of the standard “dice and fill” technique. Therefore, we developed a custom micromachining process using ultrafast lasers to fabricate the array from 480 µm‐thick single‐crystal Lead Magnesium Niobate‐Lead Titanate (PMN‐PT) (Figures S3–S5). The array face was laminated with three matching layers to form an exponentially tapered acoustic impedance profile, and the array elements were wire‐bonded to a carrier printed circuit board (PCB) (Figures 1c,d; S6–S9). To avoid fabrication challenges with the wire bonding, we chose to omit any backing layer in this work. The final array had a resonance frequency of 3.1 MHz (Figure S10) and element pitch of 500 µm, making it capable of imaging in a 57° volumetric cone beneath the array.

While the sparsity of the CODA array is advantageous in reducing system complexity, it also comes with a steep cost in signal‐to‐noise ratio (SNR) of ‐24.1 dB. This is due to the greatly reduced active area of the array, which is also reflected in the high element impedance of 4.88 kΩ (Figure S10). Fundamentally, increasing the SNR of any signal requires either increasing the signal strength or the use of averaging, both of which are problematic in pulsed ultrasound. Overcoming a ‐24.1 dB deficit would involve either a 16x increase in transmit voltage or the averaging of 256 waveforms. Such voltages are impractical and pose clear safety risks, while the acquisition and digital decimation of so many waveforms would consume excessive power for a portable device. Standard coded excitations such as binary sequences [21, 22], fast chirps [23, 24], Golay waveforms [25], and frequency‐domain schemes [26] pose similar problems in terms of power consumption since they still require high‐frequency sampling. It is important to note that wideband pulsed systems used in imaging have an inherently high per‐channel power consumption, because the entire RX signal chain must operate at or near the carrier wave frequency of the piezoelectric transducer (*f_RF_*). The analog to digital converter (ADC) in particular tends to consume a great deal of power due to sampling at typically 4*f_RF_*, or 2x the Nyquist rate, which is commonly done to allow efficient digital filtering. For reference, our CODA array would require a sampling rate of > 12.4 MHz if used with a pulsed system.

### 2.2 Chirped Data Acquisition System Design

To address the SNR challenge, we developed a new “chirped data acquisition” (cDAQ) ultrasound architecture (Figure 1e) that leverages principles of compressive sensing to boost the SNR at low system voltages while simultaneously realizing a two‐orders‐of‐magnitude reduction in sampling rate [27, 28, 29]. Fundamentally, this technique performs the signal averaging operation in analog by downconverting the received signal frequency, passively filtering, and finally sampling at a reduced rate–avoiding energy‐intensive oversampling and digital decimation altogether. As a result, while the CODA array reduces the number of power consuming RX channels by 16x, the cDAQ architecture realizes a further multiplicative reduction in per‐channel power consumption of 1.85x, resulting in a 29.6x power reduction compared to an equivalent full matrix array. By transmitting energy over a longer duration while maintaining the same peak pressure, the chirp waveform also increases the total acoustic energy delivered into tissue without exceeding voltage or pressure limits. This effectively improves SNR and enables imaging at greater depths, even under low‐voltage or safety‐constrained transmit conditions.

In the cDAQ architecture, the standard pulsed excitation is replaced with a swept‐frequency continuous wave function $f_{chirp}\;\left(t\right)=e^{2\pi i(\phi_{i}+f_{0}t+\frac{1}{2}\frac{df}{dt}t^{2})}$, defined by the initial phase angle (ϕ_*i*_), the initial frequency (*f*_0_), and the chirp ramp rate (*df*/*dt*). The complex‐valued chirp is digitally synthesized and thresholded on a field‐programmable gate array (FPGA) using a numerically controlled oscillator (NCO), producing square wave in‐phase *L* *O_I_* =  *sgn*(*R*(*f_chirp_*)) and quadrature‐phase *L* *O_Q_* =  *sgn*(*J*(*f_chirp_*)) copies of the signal. The LO_I_ signal is power boosted by half‐bridge TX drivers to produce the piezo drive signal *f_RF_* (Figure S11). The TX piezo elements then radiate the acoustic energy as diverging waves, which scatter and reflect from impedance discontinuities as they travel through the tissue volume. The reflected and time delayed echo *f_RF_* (*t* − Δ*t*) is captured by RX elements and enters the receive signal chain, first being amplified by low noise amplifiers (LNA). In the next step, the echo is demodulated by analog multiplication with the LO_I_ and LO_Q_ signals and low pass filtered (LPF), producing the intermediate frequency $f_{IF}\;\left(t\right)=e^{2\pi i(-f_{0}\Delta t+\frac{1}{2}\frac{df}{dt}\Delta t^{2}-\frac{df}{dt}t\Delta t)}$. This *f_IF_* signal has the property of translating the echo time delay (Δ*t*) into a proportional frequency ($\frac{df}{dt}\Delta t$) determined by the chirp ramp rate. Small time delays are converted to low *f_IF_* frequencies, so a programmable high pass filter (HPF) and programmable gain amplifier (PGA) are used to suppress internal array reverberations and optimize the system dynamic range. An analog to digital converter (ADC) samples the *f_IF_* signal, sending the waveform data to the FPGA. After sampling, the *f_IF_* waveforms can be efficiently transformed back into the standard pulsed format by FFT‐accelerated matched filtering, allowing the convenient use of standard beamformers. For this work, we wrote a custom GPU accelerated beamformer utilizing the “Baseband Delay‐Multiply‐and‐Sum” (BB‐DMAS) algorithm with *p*  =  2.5  [30], enabling real‐time data acquisition, beamforming, and visualization.

To showcase the capabilities of the architecture, we implemented a cDAQ (Figure 1f) with 64 TX and 64 RX channels. The transducer array, RX LNA, and TX driver were stacked vertically in a probe head to minimize capacitive loading of the RX elements (Figure 1g). The demodulators, ADCs, FPGA, and Teensy 4.1 microcontroller were integrated in the cDAQ “motherboard.” A pair of I/Q sampling, 4 MHz ADCs were configured to scan all 64 RX channels, with each channel sampled at just 124 kHz–a 100x reduction compared to the previously mentioned 12.4 MHz requirement for a pulsed system. The anti‐aliasing LPFs were set for a ‐3 dB cutoff at 45 kHz, reducing the receive signal chain to near audio frequencies. Due to the low sampling rate and reduced frequencies, the ADC power consumption was reduced to just 1.50 mW/(channel·MHz), meaning our discrete implementation is 1.85x more efficient compared to commercially available integrated circuit ultrasound chips (Figure S12 and Table S1). As the ADC typically accounts for the majority of power consumption in ultrasound receivers, this improvement represents a key advantage of the proposed architecture. To demonstrate the volumetric imaging capabilities of the full system, we used the cDAQ and CODA array to image several spiral springs (Figure 1h; Video S1) since they present a complex but easily understandable structure. Comparing the photographs against the ultrasonic images demonstrates that the CODA array can reconstruct these 3D objects with a high degree of accuracy, and shows the advantage of real‐time 3D ultrasound in visualizing and understanding sophisticated 3D structures.

### 2.3 Performance Comparison of cDAQ to Commercial Pulsed Ultrasound System

**FIGURE 2:**
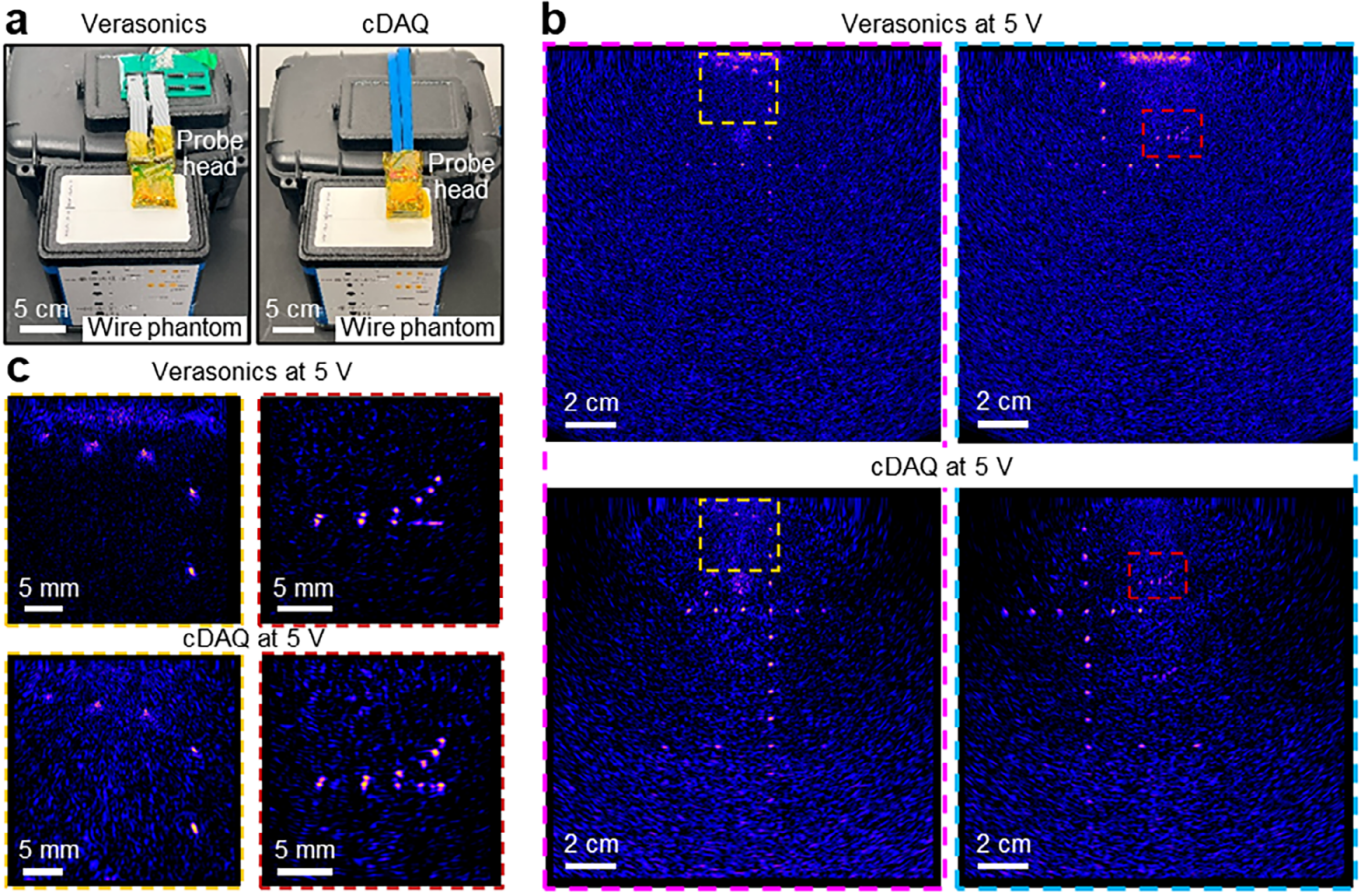
Comparison of imaging performance of the cDAQ system with the Verasonics Vantage 256 system. (a) Experimental setup for imaging of wire phantom (CIRS, model 040GSE) using Verasonics Vantage 256 system (left) and cDAQ system (right). The same 1D array was used with both systems. (b) The imaging of two different locations in a wire phantom showing near‐field (left, pink dotted box) and resolution (right, blue dotted box) targets. The top images are from the Verasonics Vantage 256 system with a driving voltage of 5 V, while the bottom images were acquired using our cDAQ system with a driving voltage of 5 V (chirp duration: 11.7 ms, bandwidth: 2–5 MHz, acquisition depth: 18 cm). (c) Zoomed images of near field (left, yellow dotted box) and resolution (right, blue dotted box) targets, demonstrating similar focusing and resolution performance with both systems. The contrast‐to‐noise ratio CNR values for the Verasonics images are 8.85 dB (left) and 10.71 dB (right), while the CNR values for the cDAQ images are 7.10 dB (left) and 9.07 dB (right).

To evaluate the performance of our cDAQ compared to a standard pulsed ultrasound system (Verasonics Vantage 256), we performed in vitro imaging on a standard tissue‐mimicking wire phantom (CIRS 040GSE) containing near‐field, resolution, contrast, and depth targets. To provide a fair comparison, we fabricated a 1D array with 300 µm element pitch and 4 MHz operating frequency, with adaptors to connect it to both the pulsed system and our cDAQ system (Figure 2a; Figure S13) in a semi‐sparse configuration (5 TX / 64 RX) to satisfy the wiring requirements of both systems. The cDAQ transmitted 5 V chirps, sweeping a 2–5 MHz bandwidth in 11.77 ms, while the pulsed system used a single‐cycle sine wave excitation at 4 MHz with voltages ranging from 5–100 V. The array was positioned to image both near‐field and resolution targets while keeping depth targets in the field of view (Figure 2b).

At 5 V the cDAQ was able to resolve wire targets at a depth of 11 cm, compared to the pulsed system at the shallower depth of 6 cm. Next, we increased the pulsed system voltage until a similar imaging depth was reached, finding that the systems matched at approximately 92 V. This technique was chosen because it compares image features with their local noise floors, making the measurement robust to differences in system behavior. This result indicates that, for the same transducer, the cDAQ configuration achieves comparable imaging depth with an effective 25.3 dB reduction in required transmit drive amplitude relative to the pulsed system (Figure S14). Meanwhile, the zoomed images of the near‐field and resolution targets (Figure 2c) indicate that the cDAQ achieves similar focusing and resolution performance. One noticeable difference in the cDAQ images is the lack of array reverberation (the bright region at the top), which is due to an algorithmic difference. As part of the cDAQ preprocessing, we measure and subtract NCO‐generated deterministic phase “noise” to prevent image degradation, which also has the effect of removing the static reverberation artifact.

### 2.4 In Vitro Characterization of CODA Array and cDAQ System

**FIGURE 3:**
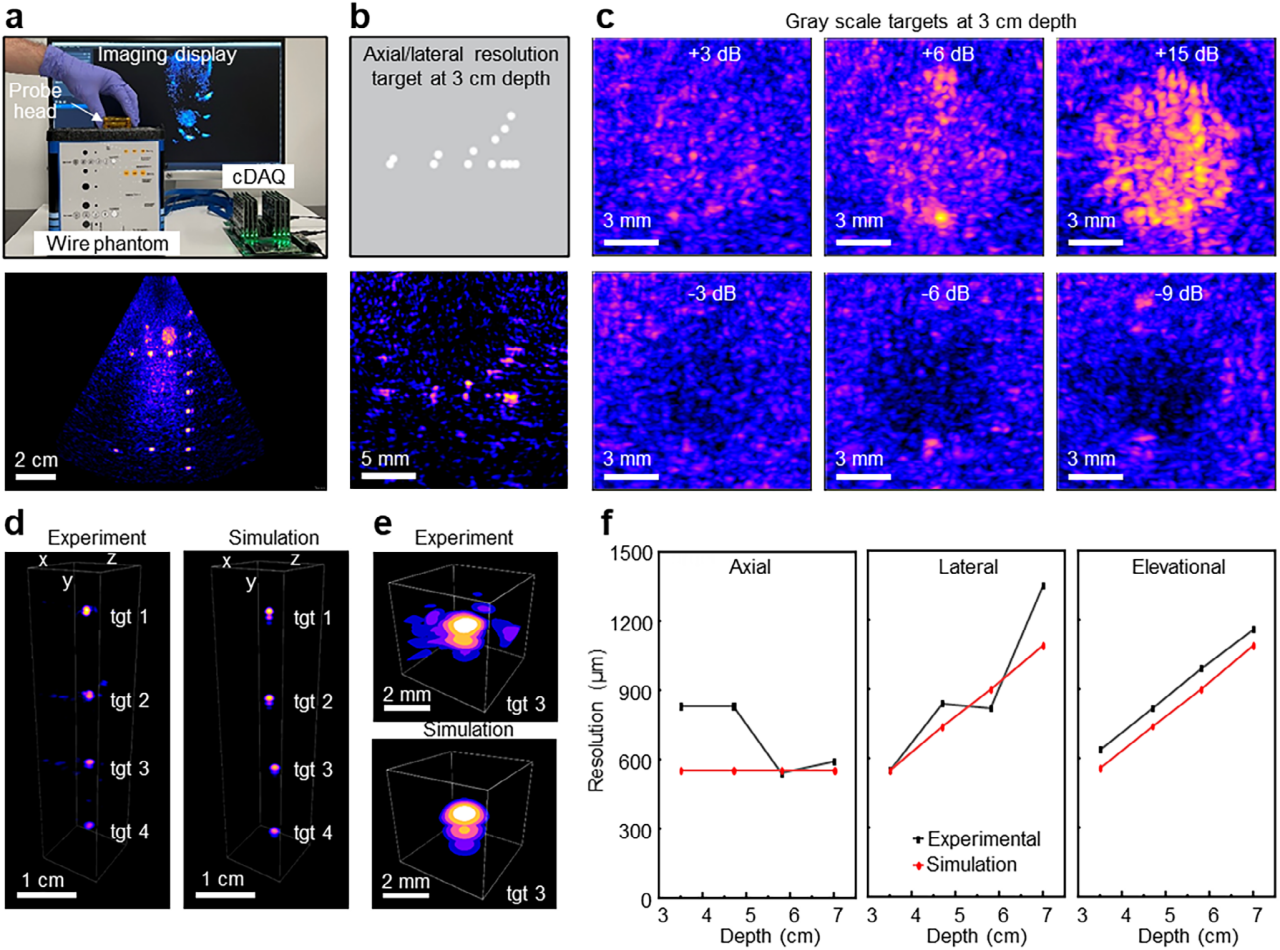
The acoustic performance of the CODA array. (a) Photograph showing the CODA array set up on the wire phantom (top) and the corresponding ultrasound image (bottom) (chirp duration: 10.9 ms, bandwidth: 2–4 MHz, acquisition depth: 25 cm), achieving an ultrasound image with 11 cm depth and a wide FOV. (b) Corresponding schematic view and ultrasound image of the resolution targets in the wire phantom at a 30 mm depth. (c) Ultrasound images of the grayscale group at 3 cm depth, featuring hyperechoic targets (+3, +6, and +15 dB) and hypoechoic targets (−3, −6, and −9 dB). The CNR values of the images for the +3, +6, +15, −3, −6, and −9 dB targets are −0.56, 1.64, 4.72, −1.72, 0.64, and 1.47 dB, respectively. (d) In vitro imaging of 250 µm spherical targets embedded in a custom gelatin phantom to measure the volumetric point spread function (PSF), with experimental results on the left and simulations on the right (chirp duration: 10.4 ms, bandwidth: 1–5 MHz, acquisition depth: 12 cm). (e) Zoomed image of a target, with the experimental result on top and simulation on the bottom. (f) PSF resolution measurements in the axial, lateral, and elevational dimensions. Black and red lines indicate experimental and simulation results, respectively.

Having compared the performance of the cDAQ and Verasonics systems on a mutually compatible 1D array, we proceeded to the in vitro characterization of the CODA array on the cDAQ system. First, we re‐imaged the wire phantom with a driving voltage of 18 V (Figure 3a, top, Video S2). The wire phantom does not contain 3D features, so we beamformed a thin slice to emulate B‐mode imaging for comparability with the earlier 1D array. The CODA array achieved deep imaging with the wire targets visible to > 11 cm, with a wide field of view of 57° (Figure 3a, bottom). The resolution targets were imaged with similar results to the 1D array (Figure 3b), although some defocusing is visible. Additionally, contrast cylinders ranging from −9 to +15 dB were used to demonstrate the contrast sensitivity of the array at a depth of 30 mm (Figure 3c). The results show that the array has sufficient contrast sensitivity to distinguish both hyperechoic and hypoechoic volumes from the surrounding tissue.

Evaluation of the volumetric imaging resolution was carried out using a custom gelatin phantom with embedded 250 µm solder balls acting as point reflectors (Figures 3d; S15, and Methods). For comparison, we simulated the imaging performance of an ideal CODA array, using the impulse response modeled using PiezoCAD (Sonic Concepts, Inc.) with the array waveforms generated by our custom simulation code [13]. The resulting experimental and simulated images (Figure 3d) are mostly in agreement, although zoomed images (Figures 3e; S16) again show a slight defocusing of the real array. Overall, the defocusing seen here and on the earlier wire phantom can likely be attributed to uneven capacitive loading of the RX piezo elements caused by unequal wire lengths in the LNA, which is more significant for the CODA array due to the high element impedances (Figure S10). There may also be micrometer‐scale bending of the array caused by the spring‐loaded pin headers used in the probe head mounting. To quantify the device's spatial resolution, we treated the point reflections as a point spread function (PSF) and measured the ‐6 dB widths on each axis (Figure 3f). As shown, the experimental measurements closely match simulation results, with the exception of the axial resolution for targets 1 and 2. This is due to a merging of the primary reflection with an internal array reverb at shallow imaging depths. Across imaging depths of 3.5–7 cm, the measured lateral, axial, and elevational resolutions ranged from 1350–550 µm, 830–540 µm, and 1160–640 µm, respectively. At an imaging depth of approximately 6 cm, the resolutions were 820 µm (lateral), 540 µm (axial), and 990 µm (elevational), in good agreement with simulation results. Figure S2 further illustrates how the PSF and spatial resolution vary across different angles, positions, and depths.

Unlike a pulsed ultrasound system, the cDAQ transmits and receives simultaneously. As a result, TX‐to‐RX crosstalk is an important parameter that determines the maximum transmit voltage the cDAQ can use without saturating the LNA. On the CODA array, the corner elements are the most susceptible to this problem. We characterized the crosstalk by measuring the signal from a corner RX element, while firing each TX element in sequence (Figure S17). The maximum crosstalk was ‐17.2 dB, which led us to select the maximum drive voltage of 18 V. Additionally, we measured the temperature rise caused by the CODA array in a tissue‐mimicking gelatin phantom by placing a miniature type‐K thermocouple beneath the array (Materials and Methods). We observed a temperature rise of 1.2°C after 30 min of operation and imaging, which is within the FDA's permissible limit [31]. Based on the pattern of temperature rise (Figure S18), most of the heating can be attributed to quiescent power dissipation in the LNA, with negligible heating caused by transmitting. This is expected since the LNA consumes 240 mW, while the peak transmit power is 13.5 mW based on the excitation voltage and element impedance (Materials and Methods, Figure S12). It is notable that the full receiver signal chain, including the parts on the cDAQ motherboard, consumes approximately 1300 mW, meaning that the transmit power is at most 1% of the system total.

### 2.5 Soft‐Tissue Imaging (Real‐Time 3D) Demonstration

**FIGURE 4:**
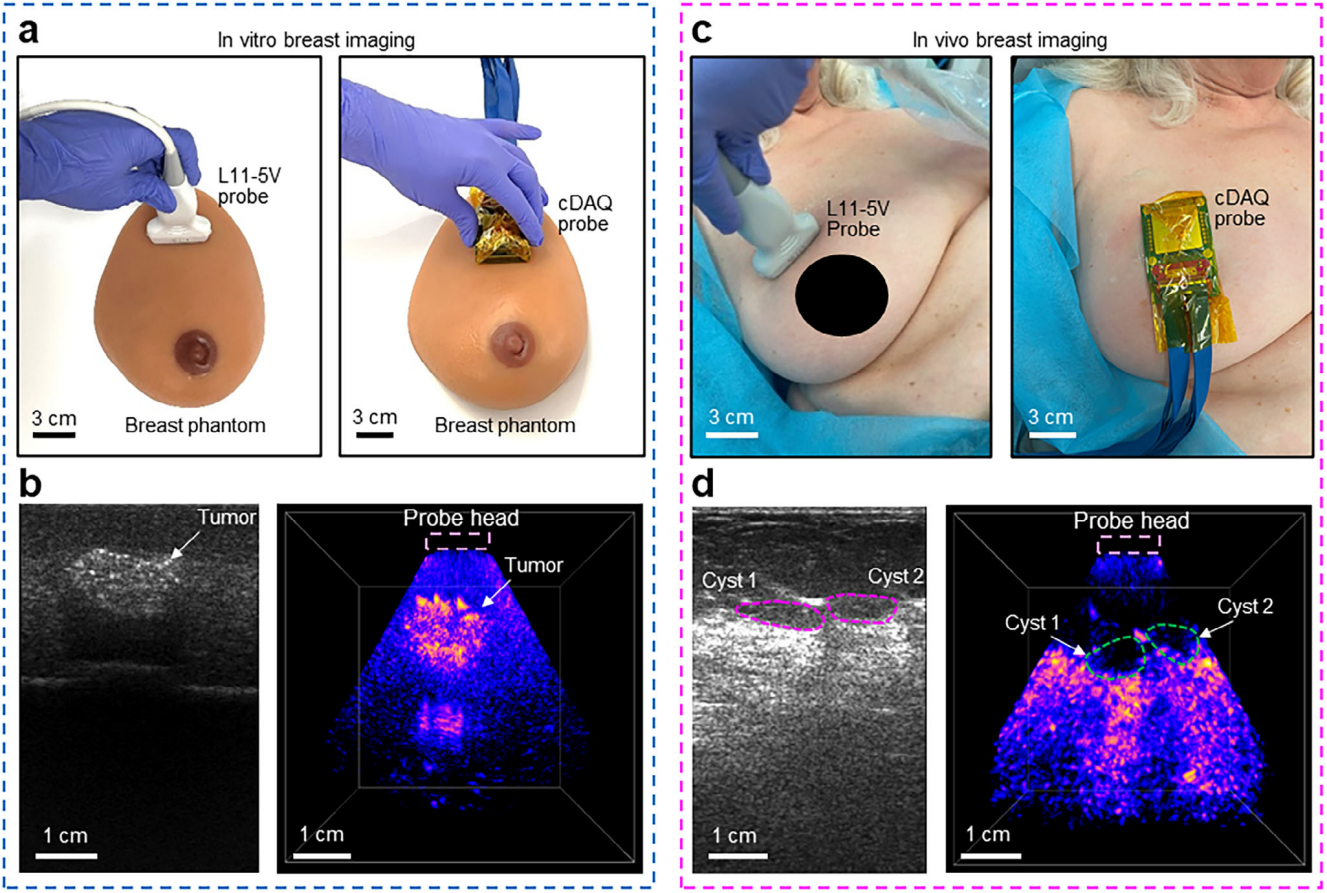
The in vitro study on breast phantom and in vivo study on breast tissue. (a) Photo of the in vitro imaging setup on a breast phantom. (b) Corresponding ultrasound images of a hyperechoic (bright) tumor in a breast phantom with a commercial L11‐5v probe attached to Verasonic 256 system (left) and a CODA array attached to the cDAQ system (right) (chirp duration: 7.8 ms, bandwidth: 1–5 MHz, acquisition depth: 8 cm). The tumor appears larger in the CODA + cDAQ image because the 4D imaging reveals the entire tumor mass at the same time. (c) Photo of the in vivo experiment, with the commercial L11‐5v probe (left) and our CODA probe head (right) attached to the subject's right breast. (d) In vivo ultrasound image of hypoechoic (dark) cysts in breast tissue taken by L11‐5v probe (left) and our CODA probe head (right) (chirp duration: 3.5 ms, bandwidth: 2–4 MHz, acquisition depth: 8 cm). The commercial probe makes the cysts appear flattened and at a shallower depth because of applied pressure and indentation of the tissue, while negligible deformation is observed using the CODA array.

Next, we proceeded to demonstrate the ability of the cDAQ and CODA system in biological tissue imaging, first by in vitro imaging of a breast tumor phantom, and second by in vivo imaging of human breast tissue. For the breast tumor phantom (Figure 4a), the cDAQ was used to capture a volumetric image of the mock tumor (Figure 4b). The Verasonic pulsed system was used with a L11‐5v linear array to capture a B‐mode image for comparison (Figure 4c). For the in vivo test, we recruited a female subject who has several fluid‐filled cysts in her right breast at a depth of approximately 2–3 cm. The same two systems and probes were used to image the cysts, with the probes placed in the same position on the breast (Figure 4d; Video S3). The images demonstrate that the cDAQ and CODA system can successfully localize the cysts. Additionally, in the image captured by the cDAQ (Figure 4d, right), the cysts appear deeper and more spherically shaped compared to the image captured by the L11‐5v (Figure 4d, left). The CODA probe causes reduced mechanical deformation of the breast tissue (Methods and Figure S19) due to its larger faceplate surface area compared to the L11‐5v probe. This is important for long term monitoring applications because placement‐induced deformation is operator dependent and degrades the ability to compare images over multiple sessions [32]. Maintaining the shape of cysts intact during imaging is crucial, particularly for applications such as breast cancer detection to quantify the progression of tumor size over time [33].

## 3 Conclusion

We have reported an end‐to‐end 4D ultrasound architecture consisting of a novel sparse array geometry and a co‐designed data acquisition system. The CODA array achieves wide‐angle (57°) imaging while reducing the number of elements from O(N^2^) to O(4N), simplifying fabrication and making high‐resolution volumetric imaging more practical. Meanwhile, the cDAQ lowers the required voltage and sampling rates by more than an order of magnitude, while reducing the per‐channel power consumption by nearly a factor of two compared to commercially available integrated circuits. Furthermore, the entire system, including the array, cables, and data acquisition electronics, weighs just 1.15 lbs in total making it a fully portable ultrasound system for higher accessibility. Systematic in vitro characterization and in vivo imaging of breast cysts demonstrate that our system is well‐suited for imaging soft tissue organs with minimal tissue distortion. Our architectural approach enables high performance, real‐time 3D ultrasound, removing a key and persistent blocker for the next generation of medical devices.

This work defines the convolutional optimally distributed array (CODA) as any array that produces a fully sampled, unity‐valued virtual aperture, and introduces the box array with separated subarrays as an optimal implementation of this principle. A broad design space of alternative geometries also satisfies the CODA condition, and a systematic exploration of their trade‐offs in sensitivity, sparsity, and reconstruction performance would be a valuable direction for future research. Further optimization of the array bandwidth and reverberation characteristics could enhance imaging performance, but will require advances in acoustic matching and backing materials that maintain the integrity of the gold nanowire interconnects between the elements and the PCB. Near‐field imaging remains limited by acoustic and electrical crosstalk, and improved shielding of exposed elements could significantly enhance image quality at shallow depths. Finally, operator dependence continues to be a major barrier to the accessibility of ultrasound imaging; integrating CODA‐based 3D systems with intelligent guidance and automation could help overcome this skill barrier and expand access to advanced diagnostic imaging.

## 4 Experimental Section/Methods

### 4.1 Fabrication of Ultrasound Arrays

We used two types of piezoelectric materials: PMN‐PT (TRS Technologies, Inc.) and PZT (Steiner & Martins, Inc.) to fabricate ultrasound arrays. The piezoelectric materials were purchased in disks 50 mm in diameter, in thicknesses of 0.48 mm for the PMN‐PT CODA array and 0.4 mm for the PZT 1D array, giving thickness mode resonances of 4 and 5 MHz, respectively. The disks were poled in the thickness direction by the manufacturer and came with screen‐printed silver electrodes on both sides. To facilitate wire bonding, 10 nm of chromium and 150 nm of gold were sputtered on the top surface of the disk. A laser cutter (microPREP PRO) was then used to cut the array outline out of the disk. The CODA array was cut with a 20.3 × 20.3 mm square outline, while the 1D array was cut to 27.8 × 11.3 mm.

After cutting, the array was inserted into a custom‐fabricated carrier PCB. A thin layer of silver conducting paint (SPI Supplies) was used to connect the bottom electrode of the array to the PCB ground layer. After the silver conducting paint was cured, the bottom surface of the array was coated with three matching layers with acoustic impedances of 15.24, 7.04, and 3.25 MRayls, respectively. These matching layers serve to facilitate wideband acoustic coupling to human tissue and reinforce the mechanical strength of the entire array.

In the next step, the array was micromachined from the top side using an ultrafast femtosecond laser system. The system was capable of outputting up to 10 W at 343 nm (UV) wavelength (Light Conversion 40‐Watt Carbide with harmonic generator) and incorporates a 300 × 300 mm stage (Griffin Motion) and galvo system (ScanLab IntelliScande‐14). This laser machining step cuts out all the transducer elements without applying significant mechanical or thermal stress to the ceramic material. We achieved approximately 40 µm kerf using fast (high‐power) laser processing settings (Figure S5) to machine our arrays. Finally, the laser was used to remove the thin electrode layer in the gap region between the edge of the piezoelectric ceramic and the elements, since this metal layer could otherwise cause short circuits with the wire bonding. In total, the machining process only took approximately 3–10 min to complete. After micromachining the array, the elements were electrically bonded to the array carrier PCB using a standard wire bonding technique (WestBond 7730).

### 4.2 Design of Chirped Data Acquisition System (cDAQ)

The Chirped Data Acquisition System (cDAQ) was designed to achieve enhanced sensitivity using chirp‐encoded excitations to counterbalance the low SNR of the sparse array, while reducing the sampling rate, voltage, and power consumption compared to conventional ultrasound imaging systems. Furthermore, the system was designed to acquire the full matrix channel data, meaning it can reproduce numerous transmit beamforming modalities (e.g., SAR, focused, plane wave, diverging, etc.) through software emulation.

As shown in Figure 1f, the cDAQ was composed of two segments, a “probe head” section and a “motherboard” section. The probe head includes the piezo carrier PCB, a 64‐channel low noise preamplifier, and a 64‐channel piezo driver. The motherboard section includes an I/O connector card, 8 demodulator modules, 2 ADC modules, an FPGA module, and a Teensy 4.1 microcontroller module. The Teensy 4.1 board provides a USB 2.0 High‐Speed port that was used to interface to an external PC.

Starting with the probe section, the piezo carrier PCB was designed with an Electroless Nickel Electroless Palladium Immersion Gold (ENEPIG) surface finish to enable wire bonding with the piezo elements. Traces fan out from the piezo bonding pads to another set of pads on the board perimeter designed as targets for spring‐loaded board‐board interconnect headers (Mill‐Max, 855‐22‐040‐30‐001101). These headers were specifically chosen for their misalignment tolerance, which was critical when using multiple separate board‐board headers.

The low noise amplifier (LNA) board stacks directly on top of the piezo carrier. The LNA was designed with a transimpedance amplifier (TIA) architecture suitable for use with the high source impedance of the small piezoelectric elements. The TIA was implemented using a decompensated op‐amp (Texas Instruments, OPA838), which provides high bandwidth (300 MHz), low noise (1.8 nV/√Hz and 1pA/√Hz), and low power consumption (1 mA). The TIA bandwidth and noise performance were highly dependent on input trace capacitance, while the high input impedance requires shielding, so the PCB layout and layer stackups were designed to support an internal signal layer with wide gaps to surrounding ground shields. Finally, the LNA board includes a low noise linear regulator (Analog Devices, LT3045) to mitigate supply drop due to cable resistance, and 50 Ω series termination resistors to isolate the op‐amps from the cable capacitance and prevent reflections.

The piezo driver board was designed as an AC‐coupled half bridge driver, which supports high frequencies, variable output drive voltages (tested to 24 V), and efficient driving. The half‐bridge uses a dual complementary bipolar transistor (ON Semiconductor, NST3946DP6T5G) in an open‐collector configuration. The input was also AC‐coupled, allowing the half‐bridge to be controlled directly by logic level signals from the FPGA. Two potential drawbacks with this design are the following: (1) It supports only 2 level square waveforms, and (2) sometimes exhibits a “runt pulse” glitch for the first few cycles of a transmit excitation. However, these drawbacks are not a problem for this system due to the demodulation scheme and long transmit waveforms.

The probe section links to the motherboard section via a pair of 1 meter flexible micro coaxial shielded cables (Samtec, 612‐HLCD‐40‐40.00‐TR‐TR‐1‐ND), which carry all TX/RX signals as well as the power supplies for the probe section. The cables connect to the motherboard via an I/O connector card, which also provides the high voltage power supply for the piezo driver board.

The motherboard itself provides interconnect and power supplies for 8 demodulator boards, 2 ADC boards, a FPGA board, and a Teensy 4.1 board. The raw power supply was sourced from a 5 V AC‐DC adaptor (Qualtek, QFWB‐30‐5‐US01), passing through a protection circuit before splitting off to the FPGA, I/O card, and Teensy boards. Three 3.3 V rails were generated by linear regulators (ON Semiconductor, NCP5501), separately powering the demodulator and ADC modules to prevent RF leakage and digital‐to‐analog crosstalk. High‐side current sense amplifiers (ON Semiconductor, NCV211R) were used with low‐valued (5‐20 mΩ) resistors to measure current flow through several branches of the power supply. Data logging of the power consumption was performed using a microcontroller (Microchip, PIC24FJ32GP202) with 12‐bit ADC, with the data exported over a universal asynchronous receiver/transmitter (UART) bus.

Each 8‐channel demodulator module accepts the amplified signals from the LNAs and multiplies them with the local oscillator (LO) signals in a fully differential I/Q passive mixer topology. The mixer was implemented using a fast‐switching analog multiplexer (Texas Instruments, TS3A5018). The output of this mixer is low‐pass filtered (LPF) close to the chip to avoid radiating high‐frequency energy due to effects such as charge injection. A programmable first‐order high‐pass filter (HPF) suppresses low‐frequency components of the signal due to array reverb and close reflectors. This was followed by a programmable gain amplifier (PGA) (Microchip, MCP6S91) with a voltage gain range of 1–32x. A second‐order active LPF was implemented (using op‐amp from Runic, RS624) in a multiple feedback topology to maintain attenuation performance even above the op‐amp feedback bandwidth. The output of the active LPF was followed by a higher‐frequency RC LPF, which serves as a charge buffer for the ADC. Overall, the signal chain has a ‐3 dB cutoff at 45 kHz, below the aliasing frequency of 62.5 kHz. The output of this I/Q signal chain feeds into 8‐1 analog multiplexers (Texas Instruments, TMUX1308) for channel selection to the ADC board. The HPF and PGA were configured by a microcontroller (Microchip, PIC16F15243), which receives commands from the Teensy 4.1 over a UART link. The analog multiplexers were controlled by the FPGA module. Finally, the onboard AC‐coupled signals float at a 1.25 V bias point sourced from the ADC module.

The low‐frequency signals from the demodulators were fed as inputs to 2 ADC modules, each of which contains a 16‐bit I/Q sampling differential ADC (Analog Devices, AD7380). The ADC supports up to 4 MSPS rate and was configured to scan across 32 I/Q channels from 4 demodulator boards, for an effective maximum rate of 125 kSPS per channel (Nyquist frequency 62.5 kHz). The ADC connects to the FPGA module over a dual SPI bus operating at 80 MHz and internally generates a 2.5 V reference, which was divided to 1.25 V, buffered by an op‐amp (Microchip, MCP6002), and used to bias the demodulator boards.

The FPGA module serves as the central timing and control unit of the system, coordinating chirp generation, ADC sampling, multiplexing, and high‐speed data buffering. The design uses a Lattice LFE5U85F device clocked from a 20 MHz reference oscillator with ±50 ppm tolerance. An on‐chip phase‐locked loop generates synchronized 320 and 80 MHz clock domains that independently drive the transmit and receive paths, ensuring deterministic timing across all acquisition stages. The chirp excitation was synthesized by time‐varying frequency control of an NCO core operating at 320 MHz with 24‐bit frequency resolution, which provides precise control of the transmit bandwidth and center frequency. On the receive side, a dedicated sampling controller manages the timing of analog multiplexer selection, precharge control, and dual 80 MHz SPI interfaces connected to two 16‐bit ADCs. The acquisition sequence was defined by an internal programmable state machine that synchronizes each conversion event with the transmit timing reference. The digitized data from both ADCs were combined into 32‐bit words and written into dual block‐RAM buffers through a custom memory interface that supports ping‐pong operation, enabling continuous acquisition while previous frames were transferred. Buffered data were streamed to the Teensy microcontroller through a 16‐bit parallel bus operating at 40 MHz, providing up to 640 megabits per second of sustained throughput. The Teensy configures and monitors the FPGA through a serial peripheral interface that accesses a register‐mapped interconnect linking all internal modules. This modular design separates the transmit, receive, and data‐transfer pipelines, allowing true parallel operation and accurate timing alignment across the system. Figure S7 presents a comprehensive overview of the imaging sequence, highlighting the coordinated operation of the individual hardware modules. The FPGA implementation was essential for combining deterministic hardware control, low‐latency streaming, and reconfigurable digital signal orchestration that would not be achievable using conventional microcontrollers or fixed‐function digital logic.

### 4.3 Imaging Algorithms for cDAQ

The data processing and image reconstruction algorithms were written in Python, leveraging the Numba and CuPy libraries to produce “just‐in‐time” compiled codes for both CPU and GPU targets. The preprocessing steps were mostly performed using parallelized CPU codes (AMD Ryzen 9 5950X), while the core beamforming algorithm was GPU‐accelerated (NVIDIA RTX 3070). After beamforming, the Napari library was used to render and display the 4D video. Our custom software was multithreaded, with the Napari rendering engine running in the main thread to enable real‐time interactivity, while the cDAQ data acquisition and beamforming ran as parallelized background threads to increase the frame rate.

The data acquisition first involves sending commands to the cDAQ, setting up the chirp and sampling parameters, and defining the size and shape of the waveform buffer. The cDAQ was typically set up to perform the entire volumetric image acquisition in one shot to avoid the overhead of multiple USB transactions. After the PC receives and unpacks an image data frame, the data acquisition thread puts the waveform data in a queue for the beamformer while simultaneously saving a compressed copy on the hard drive.

The beamformer runs in two overall steps, preprocessing and beamforming. The preprocessing takes as input the complex‐valued (I/Q) time domain waveforms of *f_IF_*. An initial optional step is background subtraction to remove deterministic “noise” caused by the phase noise of the digital NCO. Next, a time domain apodization is applied using a Hanning window. A Hamming spatial apodization is then applied to the virtual array. These apodizations serve to reduce sidelobes in the volumetric PSF. Next, the waveforms were zero‐padded and fast Fourier transformed (FFT) to obtain an interpolated frequency domain representation of the signal. The negative frequency spectrum is then subtracted from the positive frequency spectrum to remove any signals that might be caused by electromagnetic interference (EMI), for example, from nearby radio stations. Each channel was then time shifted by frequency domain multiplication, to undo the time offsets caused by the ADC channel scanning (250 ns offset per channel, for RX channels 1–32 and 33–64).

After preprocessing, the waveforms were processed by a beamforming algorithm to produce the actual images. We chose to use the “Baseband Delay‐Multiply‐and‐Sum” (BB‐DMAS) algorithm [28], which has two notable advantages. First, it has a tunable weighting for the coherence factor of the beamformer, allowing it to reproduce the behavior of “Delay and Sum” (DAS) or various orders of “Delay‐Multiply‐and‐Sum” (DMAS) by selecting the p‐value. All images in this work used *p*  =  2.5. The second advantage was that BB‐DMAS was slightly faster to compute compared to standard DMAS, which wass useful for this work since the 4D beamforming frame rate was a potential bottleneck. Our GPU‐accelerated (RTX 3070) implementation of the beamformer achieved a rate of 5.2 million voxels per second, resulting in typical frame rates of 0.25–4.0 FPS for volumetric images and 5–30 FPS for B‐mode images. The FPS range was relatively large due to the number of voxels/pixels scaling with O(N^3^) for volumetric images and O(N^2^) for B‐mode images. In synthetic aperture (SAR) beamforming, the computational load scales quadratically with the number of transmit‐receive (Tx‐Rx) element pairs. Moving from a 64 × 64 sparse box array (4096 pairs) to a full 1024 × 1024 matrix array (over 1 million pairs) increases the processing demand by a factor of 256. Since delays need to be calculated across the entire voxel volume for each Tx‐Rx pair, the per‐frame processing time increases accordingly, leading to a significant drop in frame rate. With our current implementation achieving 0.25–4.0 FPS on a 12 GB RTX 3070 GPU operating near full capacity with a sparse array, a full matrix array would yield only 0.00098–0.0156 FPS, which corresponds to one frame every 1 to 16 min. Although higher‐end GPUs could enable greater parallelization and improve frame rates, our goal was to design a cost‐effective, resource‐constrained system that remains viable for large‐scale, real‐world deployment. The overall overview of these imaging modules and data streams is shown in Figure S7.

### 4.4 Fabrication of the Matching Layers

To achieve efficient transmission of acoustic waves, we used three matching layers with ¼ λ thickness to transform the piezoceramic impedance of 30 MRayl to the 1.5 MRayl of human tissue [34]. The layers were formulated as composites of EPO‐TEK 301 epoxy, tungsten (W) powder, and zirconium dioxide (ZrO_2_) powder. The acoustic impedance, materials composition, and thickness of each matching layer are as follows: (i) first layer (15.24 MRayl, EPO‐TEK:Tungsten:ZrO_2_ = 1:4.1:1, thickness 158.4 µm), (ii) second layer (7.04 MRayl, EPO‐TEK: ZrO_2_ = 1:1.8, thickness 148.9 µm), and (iii) third layer (3.25 MRayl, EPO‐TEK: ZrO_2_ = 1:0.08, thickness 135.6 µm). The thickness of each matching layer was precisely controlled using a doctor blade, and each layer was slightly lapped as necessary to remove any bumps or blemishes on the material surface.

### 4.5 Measurement of System Power Consumption

The cDAQ power consumption was measured using high‐side current sense resistors on several branches of the power supply. The three main sections of the receive signal chain (the low noise preamplifier, demodulator card, and ADCs) were each measured separately. The power consumption was measured for the following transmit frequencies: 0, 5, 10, 15, and 20 MHz (Figure S12a).

For comparison, 40 commercial ADCs with at least 80 MSPS performance were surveyed by searching for their datasheets on Digikey. Additionally, we found specifications for 10 dedicated ultrasound analog front end chips. The datasheets reported the power consumption at a variety of sampling frequencies, so we normalized them to units of mW/(channel·MHz) (Figure S12b).

### 4.6 Characterization of the Transducer Elements

The electrical impedance of the transducer elements of the fabricated array was measured using an impedance analyzer (Agilent E4991A, Agilent Technologies, Santa Clara, CA, USA). Two key parameters, resonance frequency (f_r_) and antiresonance frequency (f_a_), can be obtained from the impedance spectrum. For acoustic testing to obtain the bandwidth, a pulse echo test was performed where transducer elements were excited using a 10 V single cycle sine wave pulse and the output was measured using a 2.0 mm needle hydrophone (NH2000, Precision Acoustics) located directly underneath the element under measurement in a water tank. The relative position between the array and the hydrophone was adjusted to achieve the strongest signal. The two‐way echo response was captured and displayed on an oscilloscope (PicoScope 5444B), while the frequency domain was calculated by fast Fourier transform (FFT). The center frequency *f_c_* and −6 dB bandwidth (%BW) were calculated by.

$$f_{c}=\frac{f_{1}+f_{2}}{2}$$

$$\%BW=100\cdot \frac{f_{2}-f_{1}}{2}$$

where *f*_1_ and *f*_2_ are the frequency when the magnitude of the FFT of the echo is −6 dB, and *f*_1_ is smaller than *f*_2_ (Figure S9).

### 4.7 Preparation of Gelatin‐Based Tissue‐Mimicking Phantom

The custom ultrasound phantom was prepared following our recent report [35]. Gelatin (Medley Hills Farm) and DI water were mixed at a 1:10 ratio and stirred thoroughly by placing the beaker on a hot plate set to 110°C. Stirring continued until the gelatin was fully dissolved. A 1 cm thick piece of ultrasound‐absorbing rubber (Precision Acoustics) was cut and placed at the bottom of a glass beaker. The gelatin solution was then poured into the beaker and cooled in a refrigerator until gelatinized.

A 250 µm tin solder ball was placed at the center of the gelatin layer, and additional gelatin solution was poured into the beaker. After cooling to gelatinize this layer, a second solder ball was placed directly on top of the first ball. This process was repeated, adding solder balls separated by approximately 1 cm of gelatin matrix, until a total of five solder balls were in place. The remaining space in the beaker was filled with the gelatin solution, and the entire beaker was cooled to complete the fabrication of the phantom. The complete fabrication process is shown in Figure S15.

### 4.8 Acoustic Imaging on Phantoms

The imaging performance of the CODA array and cDAQ system was evaluated on three types of phantoms: (i) a multipurpose, multi‐tissue ultrasound wire phantom (model 040GSE Computerized Imaging Reference Systems Inc.), (ii) the SONOtrain Breast with tumors (GT Simulators, Global Technologies), and (iii) a custom‐fabricated gelatin phantom (Figure S15). For better acoustic coupling, either ultrasound gel (Aquasonic 100 Ultrasound Transmission Gel, Parker Laboratories Inc.) or DI water was used.

### 4.9 Contrast‐to‐Noise Ratio (CNR) Calculation

CNR was measured on point targets in a CIRS phantom using paired regions of interest (ROIs) for the target and its local background. Target ROIs were circular, centered on visually identified point targets, with radii selected to capture the point spread without overlapping nearby structures. Background ROIs were either a concentric annulus or an adjacent circle of equal area, placed to avoid spillover, edges, and artifacts. ROIs intersecting phantom boundaries, inhomogeneities, or saturated pixels were redefined.

Intensities were normalized per acquisition to remove global gain or offset differences. No spatial filtering was applied prior to CNR computation. Any gamma or display‐only transforms were used solely for figure generation. All statistics were computed in double precision on raw ROI pixels.

Let the mean and standard deviation of the target and background ROIs be denoted as µ_t_, σ_t_ and µ_b_, σ_b_, respectively. The pooled noise term was calculated as:

$$\sigma_{\mathrm{rms}}=\sqrt{\left(1/2\left({\sigma_{t}}^{2}+\sigma_{b^{2}}\right)\right)}$$

The linear‐domain contrast‐to‐noise ratio was:

$${\mathrm{CNR}}_{\mathrm{lin}}=\left|\mu_{t}-\mu_{b}\right|/\sigma_{\mathrm{rms}}$$

and converted to decibels as:

$${\mathrm{CNR}}_{\mathrm{dB}}=20\mathrm{lo}g_{10}\left({\mathrm{CNR}}_{\mathrm{lin}}\right)$$

For multi‐slice or volumetric datasets, CNR was computed on projected slices. When maximum‐intensity or mean projections were displayed for visualization, the corresponding CNR values were also reported.

All computations were implemented in Python using vectorized operations, ensuring deterministic results for fixed ROI placements.

### 4.10 Temperature Measurement

To measure the heat generated by the CODA array and cDAQ system, a miniature type‐K thermocouple (Evolution Sensors, Type K with bead diameter ∼300 µm) was placed on top of the gelatin phantom directly underneath the CODA array. The array was operated at full power (18 V) continuously and the temperature change was recorded for several different operating conditions (Figure S18). The temperature data was logged using proprietary software (DATAQ Instruments Hardware Manager).

### 4.11 In Vivo Clinical Study

All procedures involving human subjects with a history of breast anomalies followed the approved experimental protocol by the Committee on the Use of Humans as Experimental Subjects at MIT (COUHES, Protocol #: 2011000271A007), including the participant's completion of an informed consent form. The inclusion criteria were female gender, age between 18 and 85 years, and a body mass index (BMI) of 17 to 40 kg/m^2^. The subject did not have any significant health problems (e.g., chronic or acute cardiovascular diseases and skin diseases) or physical and/or behavioral health disabilities that limited their ability to adhere to provided instructions and complete research‐related activities.

The clinical study was conducted at the MIT Center for Clinical and Translational Research (CCTR). Imaging was performed using our cDAQ and CODA array and cross‐validated with the Vantage 256 system using a linear probe (Verasonics, L11‐5v). Acoustic coupling was achieved using Scan 11‐08 Ultrasound Gel (Parker Laboratories Inc.). The study was assisted by two clinical research nurse coordinators from the MIT CCTR.

The subject in this study was 71 years old with a BMI of 37 kg/m^2^ and a history of abnormal breast conditions. It was worth mentioning that we performed the in vivo imaging of the same subject that we imaged in our previous study [12] to observe the progression of breast cyst changes over time. The subject lay supine on an examination table and adjusted her clothing to allow ultrasound access to her breast. After applying the ultrasound coupling gel, the probe was manually positioned over the region of interest. The right breast was scanned until the cysts were located, then the target location was marked and imaged using both systems. The current prototype was hand‐held, not mechanically fixed, although its flat form factor and wide field of view reduce sensitivity to small placement errors. After probe placement, the acquisition of diagnostically useful 3D images typically required less than 1 min, including minor adjustments. Once positioned in the marked location, volumetric imaging was performed in real‐time without further probe manipulation. Finally, the ultrasound gel was cleaned from the subject's skin with a clean wipe.

### 4.12 Evaluation of Tissue Compression on a Breast Phantom

A breast‐mimicking phantom with an embedded tumor was mounted on a force‐sensing stage such that the tumor faced upward toward the probe. Custom 3D‐printed attachments were used to secure both the CODA box‐array probe and a commercial probe (Verasonics L11‐5 V imaging array) to a *z*‐axis manipulator for controlled motion over the tumor. For each probe, acoustic gel was applied at the imaging site, and the *z*‐axis manipulator was incrementally lowered to record images corresponding to forces from 0 to 11 N (Figure S19). To compare the two probes under equivalent conditions, 3D volumetric data from the CODA probe were projected into 2D by retaining the maximum intensity value along each axial line within the viewing window. A consistent region of interest (ROI) encompassing the tumor was defined for both datasets. The brightest pixels within the ROI (above the 95th percentile) were tracked across force levels to assess the apparent displacement and compression of the tumor as contact force increased. In the 2D probe images, the tumor could be clearly seen moving upward as the contact force increases, indicating substantial tissue compression. In contrast, in the 3D cDAQ images, tumor displacement was not apparent, suggesting that far less tissue compression occurred along the direction of applied force.

## Author Contributions

C.M. and C.D. conceptualized the project. C.M., A.S., M.O.G.N., and J.H. developed the laser fabrication process. C.M. and M.O.G.N. designed the ultrasound transducers and composite materials. M.O.G.N. fabricated the transducers. C.M. designed and fabricated the chirped data acquisition system. C.M. wrote the HDL code and firmware running on the FPGA and microcontrollers. C.M. wrote the software for data collection, GPU‐accelerated beamforming, and 3D visualization. SV developed the algorithm to calculate CNR. M.O.G.N. modeled the transducer impulse response, and CM performed the 3D imaging simulations. C.M. and M.O.G.N. performed the in vitro system characterization and imaging on phantoms. C.D. planned the human subject trials. C.M., M.O.G.N., A.S., and C.D. performed the in vivo breast tissue imaging. M.E., S.V., and M.O.G.N. performed the tissue deformation evaluation. C.M., M.O.G.N., A.S., J.H., S.V., D.S., A.C., T.O., C.D. contributed to data analysis and data interpretation. C.M. and M.O.G.N. wrote the paper, and all authors contributed to the review and editing. C.M., M.O.G.N., A.S., and J.H. prepared the figures, and C.M. prepared the videos. C.D. directed all the research activities and supervised the project.

## Funding

This work was supported by NSF CAREER: Conformable Piezoelectrics for Soft Tissue Imaging (grant no. 2044688), 3M Non‐Tenured Faculty Award, The Lyda Hill Foundation and MIT Media Lab Consortium funding.

## Conflicts of Interest

The authors declare no conflict of interest.

## Supporting information




**Supporting File 1**: adhm70859‐sup‐0001‐SuppMat.pdf.

**Supporting File 2**: adhm70859‐sup‐0002‐VideoS1.mp4.

**Supporting File 3**: adhm70859‐sup‐0003‐VideoS2.mp4.

**Supporting File 4**: adhm70859‐sup‐0004‐VideoS3.mp4.

## Data Availability

The data that support the findings of this study are available from the corresponding author upon reasonable request.
