# Supplementary material for: Real‐Time 3D Ultrasound Imaging with an Ultra‐Sparse, Low Power Architecture
Source: Adv Healthc Mater. 2026 Jan 29;15(16):e05310. doi: 10.1002/adhm.202505310 (PMC13107935; doi:10.1002/adhm.202505310)
Supplement: Supplementary file 1 — Supporting File 1: adhm70859‐sup‐0001‐SuppMat.pdf. [file ADHM-15-0-s004.pdf]

Supporting Information

Real-time 3D Ultrasound Imaging with an Ultra-Sparse, Low Power Architecture

Colin Marcus†, Md Osman Goni Nayeem†, Aastha Shah, Jason Hou, Shrihari Viswanath, Maya Eusebio, David Sadat, Anantha P. Chandrakasan, Tolga Ozmen, Canan Dagdeviren*

This supplementary information contains:

Supplementary Notes 1-5, Supplementary Figures 1-19, list of Supplementary Videos 1-3, and Supplementary Table 1

Supplementary Note 1: Choice of Array Design

Box-shaped and cross (X)-shaped CODA arrays are among the most widely adopted geometries in prior studies. In addition, alternative configurations such as spiral and fractal arrays have been explored to improve spatial coverage and suppress sidelobes (ref 17, main text). Among these, the box array offers notable advantages due to its structural simplicity and its ability to achieve spatial Nyquist sampling without redundancy. This property makes it particularly suitable for sparse array imaging, enabling wide field-of-view acquisition with minimal aliasing and a substantially reduced number of channels.

The box geometry also confers practical benefits for probe integration. It supports a straightforward fan-out of PCB traces, which facilitates the compact integration of transmit excitation circuitry and receive preamplifiers within the probe head. The rectangular form factor enables a simple PCB cutout for mounting piezoelectric elements, streamlining the wire bonding process. With bonding pads located in close proximity to the elements, wire bonds are shorter and more robust, thereby enhancing manufacturing yield and long-term device reliability.

Supplementary Note 2: Array Architecture Comparison and SNR Compensation

Schematic illustrations of the full-matrix, row–column, and proposed sparse box array architectures are shown below.

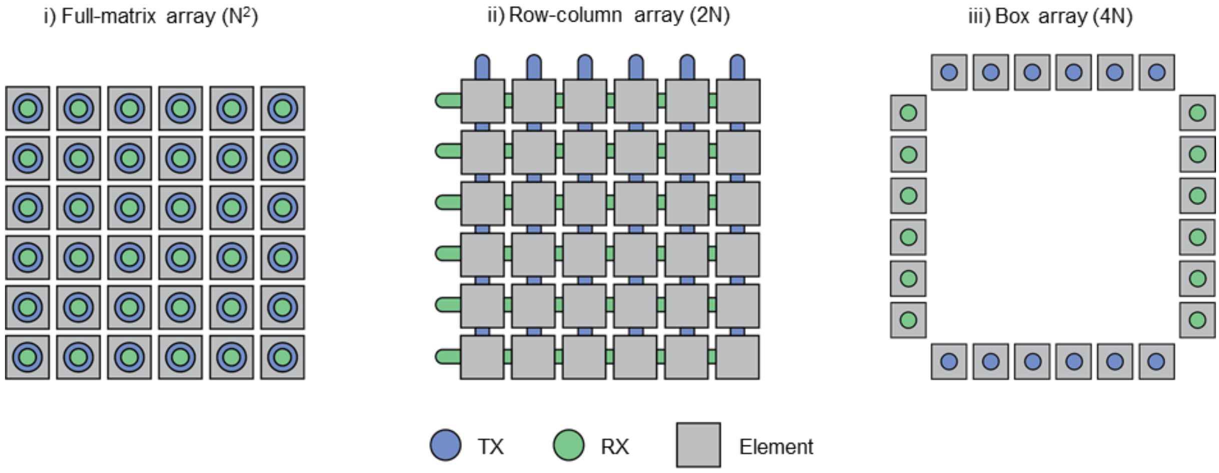

A comparison of the proposed box array with conventional full matrix and row-column architectures is summarized in the table below. The box array offers a favorable trade-off by enabling wide field-of-view imaging with a low channel count (4N), reducing the number of receive channels by a factor of 16 compared with a full matrix array (N^2). However, as

indicated in the table, this sparsity inherently limits the transmitted energy and reduces the intrinsic signal-to-noise ratio (SNR). To mitigate this limitation, we employ a long-duration chirp instead of a short transmit pulse, thereby increasing the total transmitted energy and improving the SNR at low drive voltages. This approach enables recovery of image quality that would otherwise be degraded by the sparse array configuration.

	Full matrix array	Row-column array	Box array
Physical layout	Fully populated grid of elements [c]	Two orthogonal 1D arrays [a]	Elements located on the perimeter [f, g]
Channel count	Very high (N^2) [c]	Low (2N) [a, c]	Low (4N) [f]
Field of view	Wide [c]	Narrow [a, b]	Wide [d, f]
Image resolution	Gold standard with low side lobes [c]	Good in focal region; anisotropic PSF off axis [b]	Good main lobe width and low side lobes with apodization [e, f]
Image quality	High (high SNR and low clutter) [c]	Moderate (artifacts can reduce contrast) [a, b]	Moderate to Low (energy loss due to missing elements) [d, f]
Co-Array (effective aperture)	Highly redundant filled grid (high SNR) [d]	Cross shape (missing corners in co-array) [a]	Filled box (synthesized via convolution) [f, g]
System complexity	High (requires massive cabling or ASICs for micro beamforming) [c]	Low (simple interconnects; compatible with standard scanners) [a]	Low to medium (simple interconnect but advanced signal processing) [f, g]
Primary limitation	Cost, complexity, power consumption, and data bandwidth	Limited field of view, anisotropic resolution and artifacts	Reduced contrast and SNR, and complex signal processing

References:

a. J. A. Jensen et al., "Anatomic and Functional Imaging Using Row–Column Arrays," in IEEE Transactions on Ultrasonics, Ferroelectrics, and Frequency Control, vol. 69, no. 10, pp. 2722-2738, Oct. 2022, doi: 10.1109/TUFFC.2022.3191391.

b. M. F. Rasmussen and J. A. Jensen, "3-D ultrasound imaging performance of a row-column addressed 2-D array transducer: A measurement study," 2013 IEEE International Ultrasonics Symposium (IUS), Prague, Czech Republic, 2013, pp. 1460-1463, doi: 10.1109/ULTSYM.2013.0370.
c. C. E. Morton and G. R. Lockwood, "Theoretical assessment of a crossed electrode 2-D array for 3-D imaging," IEEE Symposium on Ultrasonics, 2003, Honolulu, HI, USA, 2003, pp. 968-971 Vol.1, doi: 10.1109/ULTSYM.2003.1293560.
d. M. Karaman, Pai-Chi Li and M. O'Donnell, "Synthetic aperture imaging for small scale systems," in IEEE Transactions on Ultrasonics, Ferroelectrics, and Frequency Control, vol. 42, no. 3, pp. 429-442, May 1995, doi: 10.1109/58.384453.
e. A. Austeng and S. Holm, "Sparse 2-D arrays for 3-D phased array imaging - design methods," in IEEE Transactions on Ultrasonics, Ferroelectrics, and Frequency Control, vol. 49, no. 8, pp. 1073-1086, Aug. 2002, doi: 10.1109/TUFFC.2002.1026019.
f. R. Cohen and Y. C. Eldar, "Sparse Convolutional Beamforming for Ultrasound Imaging," in IEEE Transactions on Ultrasonics, Ferroelectrics, and Frequency Control, vol. 65, no. 12, pp. 2390-2406, Dec. 2018, doi: 10.1109/TUFFC.2018.2874256.
g. R. Cohen and Y. C. Eldar, "Optimized Sparse Array Design Based on the Sum Coarray," 2018 IEEE International Conference on Acoustics, Speech and Signal Processing (ICASSP), Calgary, AB, Canada, 2018, pp. 3340-3343, doi: 10.1109/ICASSP.2018.8462382.

Supplementary Note 3: Chirp Signal and Conversion to Beamforming Format

The chirp excitation is a continuous wave signal where the frequency ramps linearly over time. The equation for a chirp in linear units is:

$$LO(t) = e^{2\pi i\left(\phi_i + f_0 t + \frac{1}{2}\frac{df}{dt}t^2\right)}$$

where:

ϕ_i ≔ initial phase angle (0 - 1)

f_0, f_1 ≔ initial and final frequencies (Hz)

$\frac{df}{dt} = \frac{f_1 - f_0}{\tau}$ ≔ chirp ramp rate (Hz/s)

τ ≔ chirp duration (s)

Using the LO function as the excitation, the echo signal received by the RX elements will be:

$$RF(t) = LO(t - \Delta t)$$
$$= e^{2\pi i\left(\phi_i + f_0(t-\Delta t) + \frac{1}{2}\frac{df}{dt}(t-\Delta t)^2\right)}$$

where:

$\Delta t = \left(\frac{2d}{c}\right) \coloneqq$ reflection round-trip time delay (s)

$d \coloneqq$ distance to the reflector (m)

$c \coloneqq$ speed of sound (m/s)

In the cDAQ demodulators, the echo signal is multiplied with the complex valued LO signal, producing the intermediate frequency (IF) signal as follows:

$$IF(t) = LO(t)RF(t)$$
$$= e^{2\pi i\left(\phi_i + f_0 t + \frac{1}{2}\frac{df}{dt}t^2\right)} \cdot e^{2\pi i\left(\phi_i + f_0(t-\Delta t) + \frac{1}{2}\frac{df}{dt}(t-\Delta t)^2\right)}$$
$$= e^{2\pi i\left(2\phi_i + 2f_0 t + \frac{df}{dt}t^2 - f_0\Delta t + \frac{1}{2}\frac{df}{dt}\Delta t^2 - \frac{df}{dt}t\Delta t\right)}$$

As shown, the IF signal contains a large number of terms. The $2\phi_i$ term is a static phase shift depending on the initial phase angle of the LO signal. The $2f_0t$ and $\frac{df}{dt}t^2$ terms are high frequencies that do not depend on Δt and thus contain no image information – both of these are filtered out by analog low pass filters in the cDAQ hardware.

Ignoring the static phase offset and assuming low pass filtering, we are left with the parts that depend on Δt and are thus relevant to the image beamforming:

$$\mathrm{IF}(t) = e^{2\pi i\left(-f_0\Delta t + \frac{1}{2}\frac{df}{dt}\Delta t^2 - \frac{df}{dt}t\Delta t\right)}$$

This is the part of the signal that is acquired by the ADC and contains the image information. Note that the information consists of a static phase shift and a frequency, both of which are functions of Δt. This is a frequency domain signal, not a time domain signal as in standard pulsed or fast-chirped ultrasound.

Before beamforming, we must convert this signal into a format that can be processed by a standard beamformer. To do this, we use matched filtering using the equation for $\mathrm{IF(t)}$ to recover the signal contribution to a single image voxel, denoted as $\mathrm{V(\Delta t)}$:

$$V(\Delta t) = \sum_{t=0}^{t_f} f(t) IF(t, \Delta t)$$

$$= \sum_{t=0}^{t_f} f(t) e^{2\pi i\left(-f_0 \Delta t + \frac{1df}{2dt}\Delta t^2 - \frac{df}{dt} t\Delta t\right)}$$

$$= e^{2\pi i\left(-f_0 \Delta t + \frac{1df}{2dt}\Delta t^2\right)} \cdot \sum_{t=0}^{t_f} f(t) e^{-2\pi i \frac{df}{dt} t\Delta t}$$

Breaking up the equation as shown makes it clear that this conversion can be interpreted as a static phase shift coefficient multiplied by a Fourier transform of the signal $\mathrm{f(t)}$ for the reflection frequency $\frac{df}{dt}\Delta t$.

Rather than calculate the Fourier transform individually for each voxel, it is far more efficient to simply convert the entire $IF(t)$ waveform into the pulsed waveform equivalent $IF_F(f)$ using a Fast Fourier Transform (FFT). In this work, we used the following flow (in Python):

1a. Convert $IF(t)$ into $IF_F(f)$ by taking the FFT along the time axis.
1b. Generate a 1D array containing the frequency map of $IF_F(f)$ using np.fft.fftfreq().
2. Using the frequency map array, calculate the static phase shift coefficients and multiply them elementwise with $IF_F(f)$.

After completing these operations, $IF_F(f)$ is now a complex valued echo signal that can be directly used with standard time domain beamformers, simply by replacing the standard Δt indexing with $\frac{df}{dt}\Delta t$.

Supplementary Note 4: Generalized p-th root BB-DMAS Beamforming

Baseband DMAS (BB-DMAS) beamforming has several advantages compared to the more well-known DMAS algorithms. In standard DMAS the signal frequency is upconverted, in turn requiring an increased sampling frequency to meet Nyquist constraints – with proportionally increased compute and memory costs. Additionally, after beamforming the image must be bandpass filtered and enveloped to obtain the final image. The bandpass filtering can sometimes introduce ringing artifacts and a slight loss of resolution.

BBDMAS avoids both of these steps and allows beamforming to proceed at the same spatial resolution as standard DAS, but with equivalent results to DMAS. Thus, the runtime is substantially faster. Additionally, the tunable p-value of the algorithm allows greater flexibility compared to other algorithms - for example, a p-value of 1.0 is equivalent to the DAS algorithm while 2.0 is equivalent to DMAS.

In BB-DMAS the value of a voxel is calculated as follows:

$$y_{\text{BB-DMAS-p}} = \left(\frac{1}{nm} \sum_{n=0}^{N_{TX}} \sum_{m=0}^{N_{RX}} a_{nm}^{1/p} \right)^{p}$$

where a_{nm} is the appropriately time-delayed sample for the waveform from the nth/mth TX/RX channel pair.

Supplementary Note 5: cDAQ Frame Rate Calculation

To find the cDAQ system parameters the sampling rate of the ADC and the image parameters (depth and resolution) are specified first, then the chirp excitation parameters are calculated.

The input parameters are:

f_{min}, f_{max} ≔ minimum and maximum frequency limits of the chirp (Hz)

f_s ≔ ADC sampling rate (Hz)

d ≔ desired imaging depth (m)

c ≔ speed of sound (m/s)

From these we can calculate some additional basic parameters:

$f_{nyq} = f_s/2$ ≔ nyquist frequency (Hz)

$d_{rt} = 2d$ ≔ round-trip distance (m)

The chirp parameters are calculated as:

$\frac{df}{dt} = \frac{f_{nyq}c}{d_{rt}}$ ≔ chirp ramp rate (Hz/s)

$\tau = \frac{f_{max}-f_{min}}{\frac{df}{dt}}$ ≔ chirp duration (s)

The number of ADC samples and associated spatial resolution are calculated as:

$N_s = \tau f_s$ ≔ number of ADC samples

$\Delta x = \frac{d}{N_s/2}$ ≔ spatial resolution (m)

It is notable that here the spatial resolution is effectively defined as the diffraction limit emerging naturally from the behavior of the Fourier transform.

Finally, the system frame rate depends on the chirp period and the number of transmitted chirps N_{TX} required for a full image acquisition:

$\tau_{acq} = \tau N_{TX}$ ≔ full frame acquisition time

$1/\tau_{acq} = \frac{f_s c}{4N_{TX}d(f_{max}-f_{min})}$ ≔ frame rate

The following is an example calculation for the cDAQ system. The sampling rate is set to 124 kHz, with a chirp sweeping 2-5 MHz, with the max depth set to 8 cm, the speed of sound is 1540 m/s, and 64 transmit elements are used in a differential transmit mode (2 transmits per element). In this case, the number of ADC samples is 623, the frame rate is 1.6 FPS, and the spatial resolution is 257 μm.

As can be seen in the last equation, the frame rate is determined by several parameters, including the imaging depth, the bandwidth (which determines the imaging resolution), and the ADC sampling rate. This makes the cDAQ system highly configurable. In cases like this work where a high FPS is not required, the ADCs can be scaled down to reduce cost, complexity, and power consumption. Alternatively, more and faster ADCs can be used to achieve high frame rates comparable to commercial systems. For example, under the same parameters a sampling rate of 2 MHz would achieve 25.1 FPS.

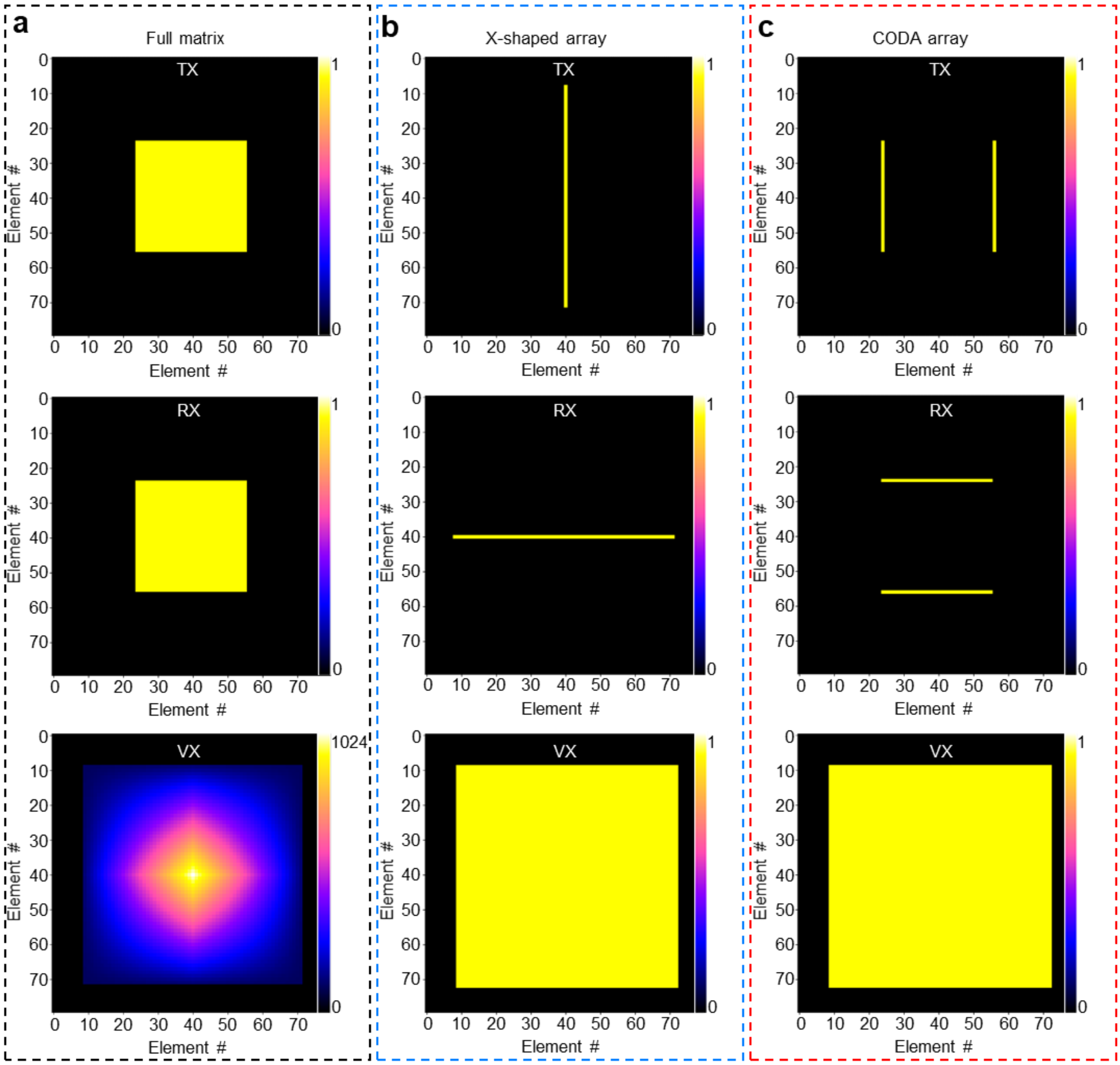

Figure S1

Virtual apertures produced by different array geometries. a) Full matrix with 1024 elements. All elements are used for both transmit and receive. The resulting virtual array (VX) is inefficient, with the center oversampled by a factor of 1024x. The values for the VX image were slightly re-scaled to make the low valued edges more visible. b) X-shaped CODA array with 128 elements (64 TX and 64 RX). This array gives a unity-valued VX array. c) Box-shaped CODA array used in this work, also with 128 elements (64 TX and 64 RX). The VX array is unity-valued. This array has the advantage of being physically smaller than the X-shaped array.

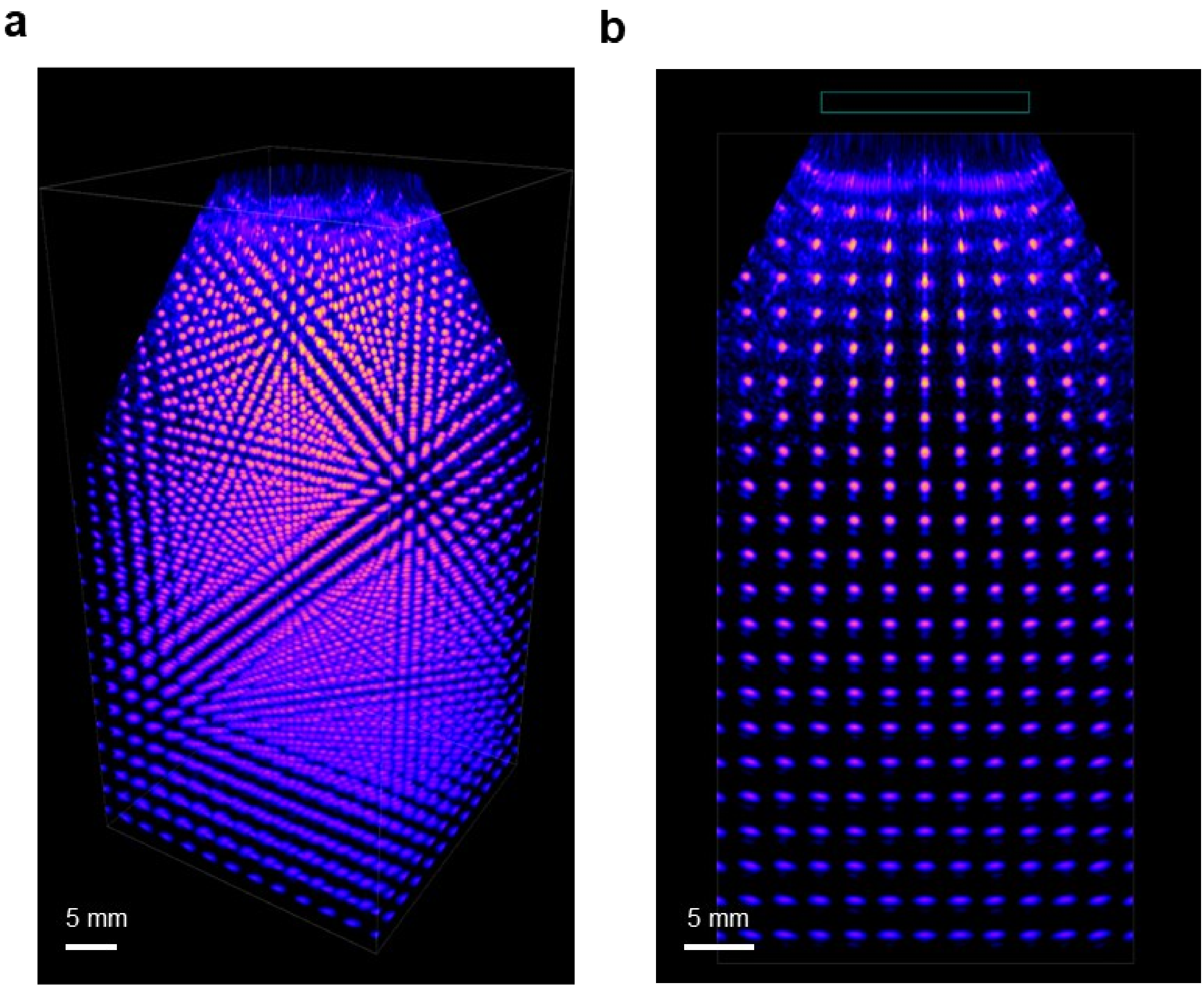

Figure S2

Simulation of the CODA Array and cDAQ system imaging a 3D grid of reflectors with 2.5 mm spacing. a) The reconstructed image in volumetric 3D. The vertical height of the image is 6 cm. The simulation models the array behavior including the experimentally measured impulse response, cDAQ analog signal processing, and the BB-DMAS beamformer. b) The same image from the side in orthographic projection. The extreme near field performance (within approx. 1 cm of the array) is visibly degraded, with reduced sensitivity and contrast.

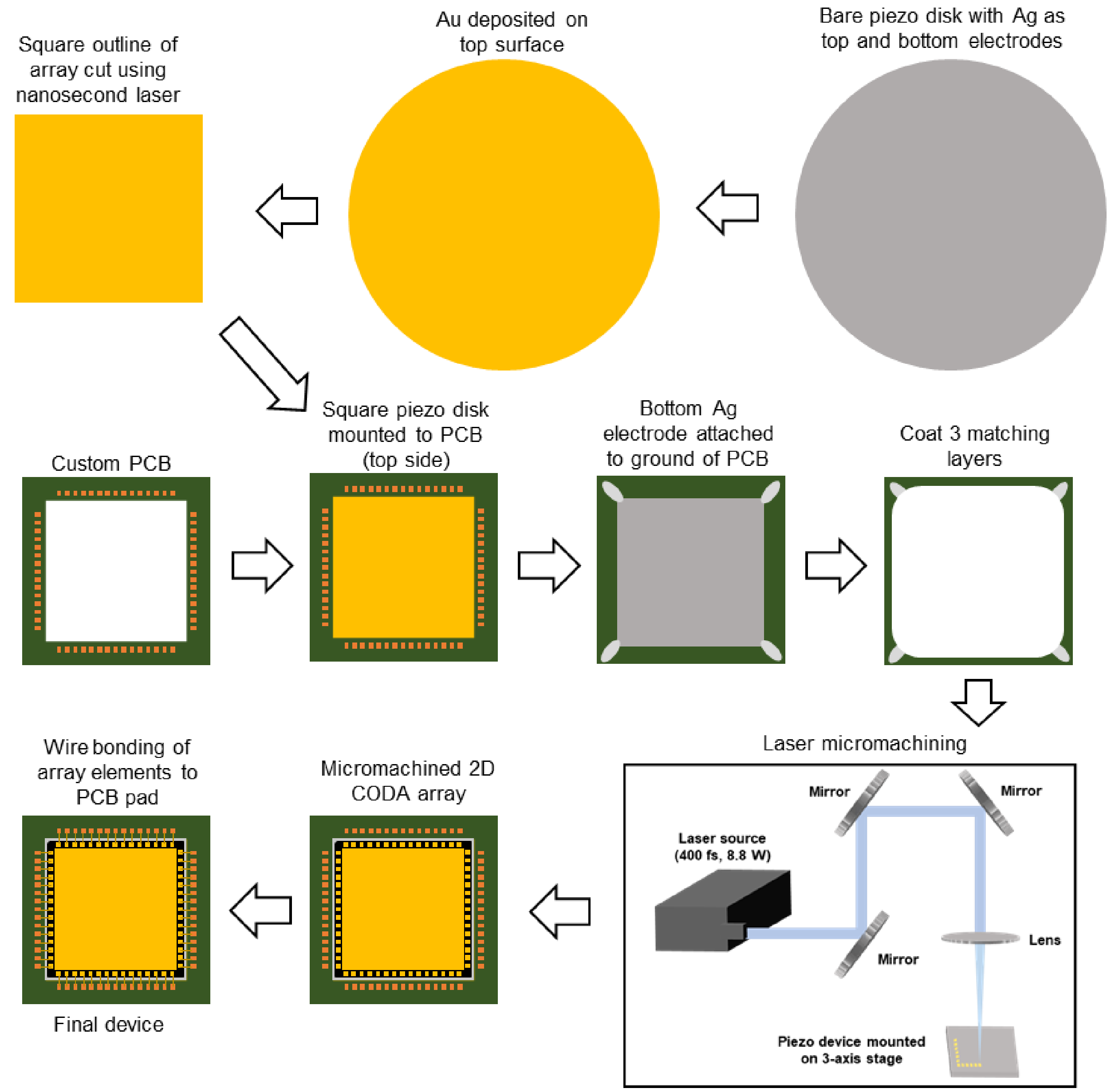

Figure S3

Fabrication process flow of CODA ultrasound array.

Fabrication starts with a commercially sourced piezoelectric disk. Laser micromachining is employed to cut the discs into the desired pattern to form the array. Finally, gold (Au) wire bonding connects each element of the array to the carrier PCB for interfacing with the data acquisition electronics. This laser micromachining process reduced the total array production time down to 10-15 minutes and enabled 100% yield in array fabrication.

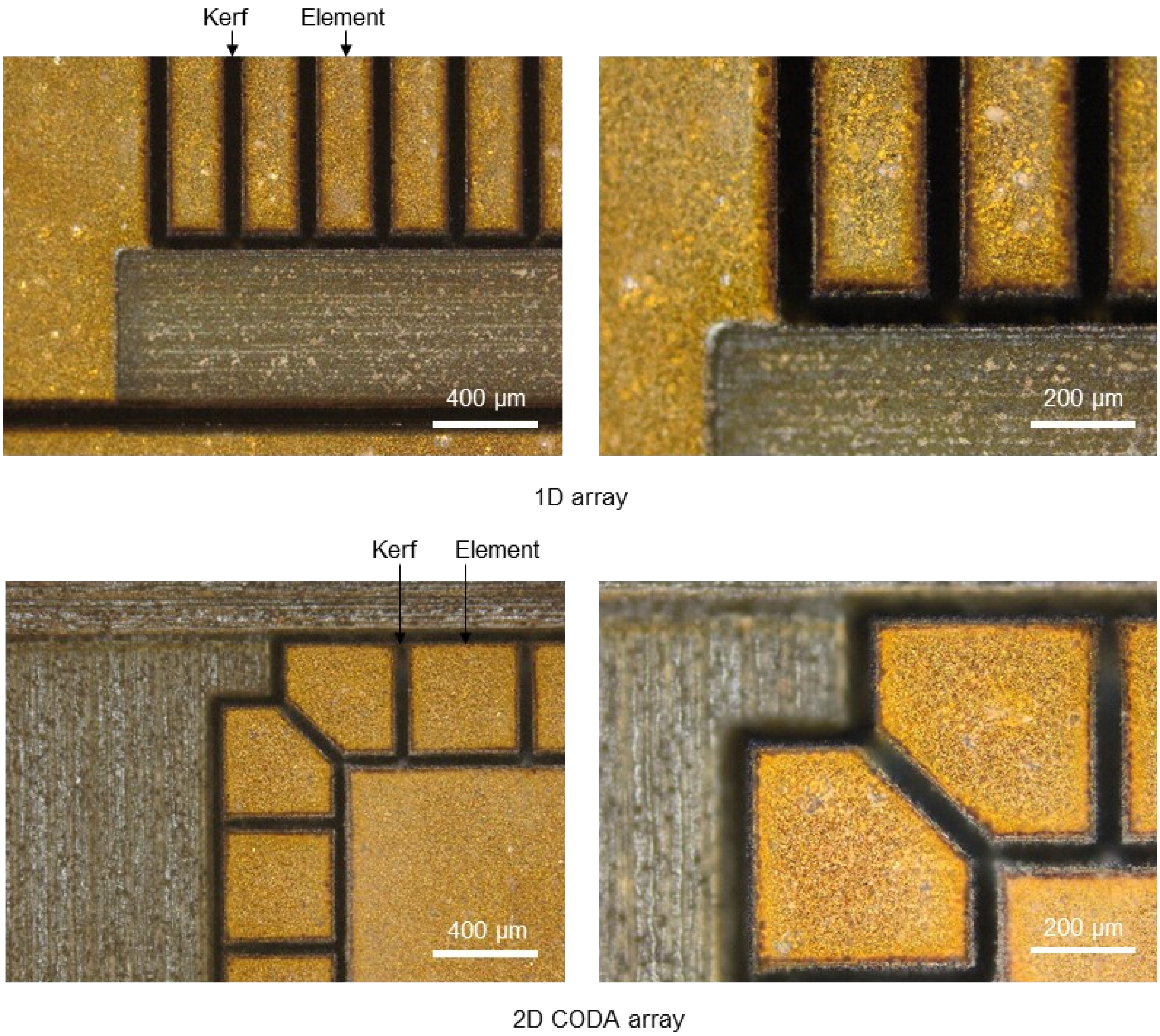

Figure S4

Microscopic image of 1D array (top) and 2D CODA ultrasound array (bottom).

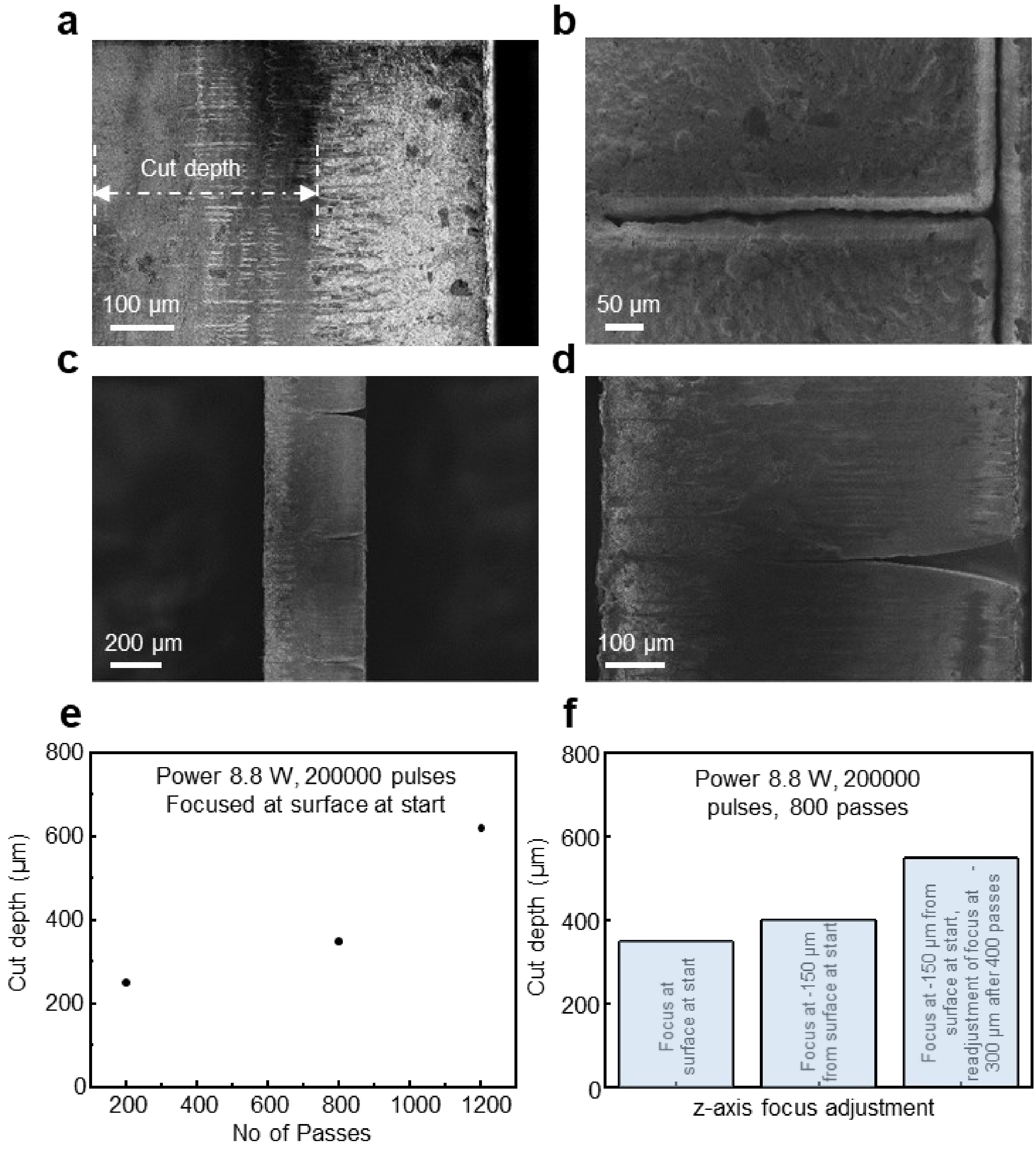

Figure S5

Laser micromachining process optimization. Scanning electron microscopy (SEM) images showing laser micromachining of PZT. a) The surface where the laser is irradiated shows a smooth cut (machining condition: Power 8.8 W, 200000 pulses, 800 passes, focus at the top surface). b) Top view of the cut showing the kerf. c) Cross-sectional view of the cut. d) Magnified view of the cross section. e) Cut depth versus number of laser passes f) Cut depth versus z-axis focus adjustment.

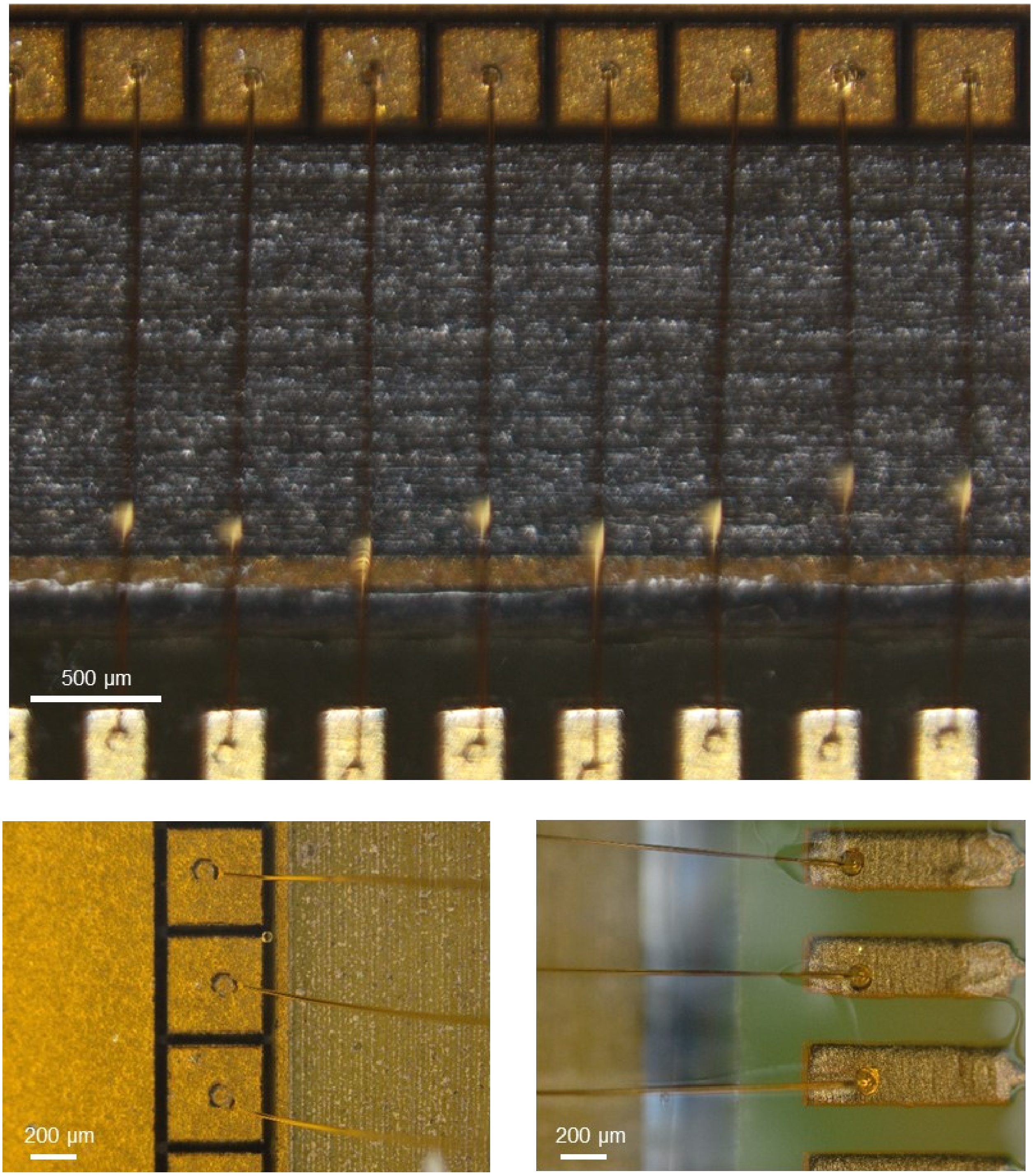

Figure S6

Wire bonding to connect the top electrode of each element to the contact pads on the PCB.

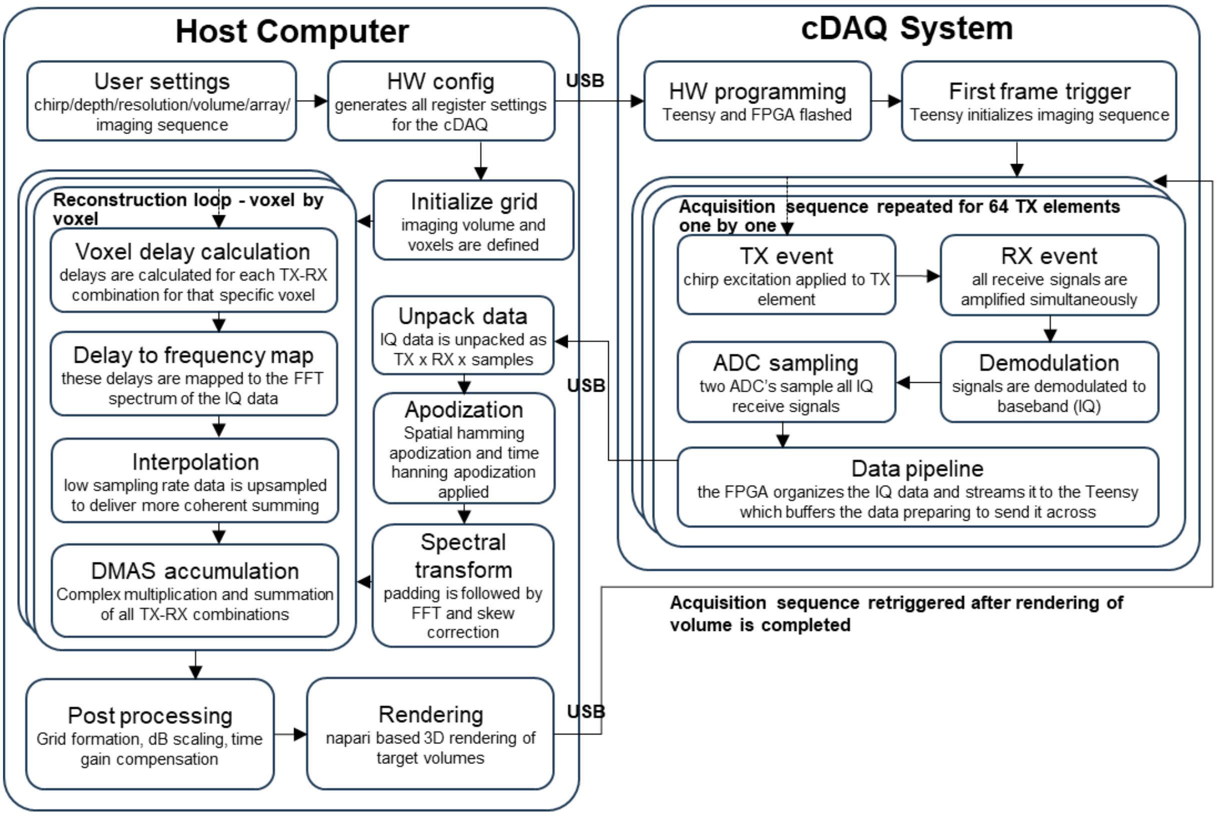

Figure S7

System architecture and imaging workflow. The workflow integrates host computer control with cDAQ hardware acquisition. The sequence begins with hardware programming followed by sequential data collection across 64 transmit elements. Raw IQ data is processed on the host through apodization and spectral transforms. Reconstruction occurs via a voxel-by-voxel loop utilizing delay multiply and sum (DMAS) accumulation. The final 3D volume is

rendered in Napari, with completion retriggering the acquisition loop for continuous imaging.

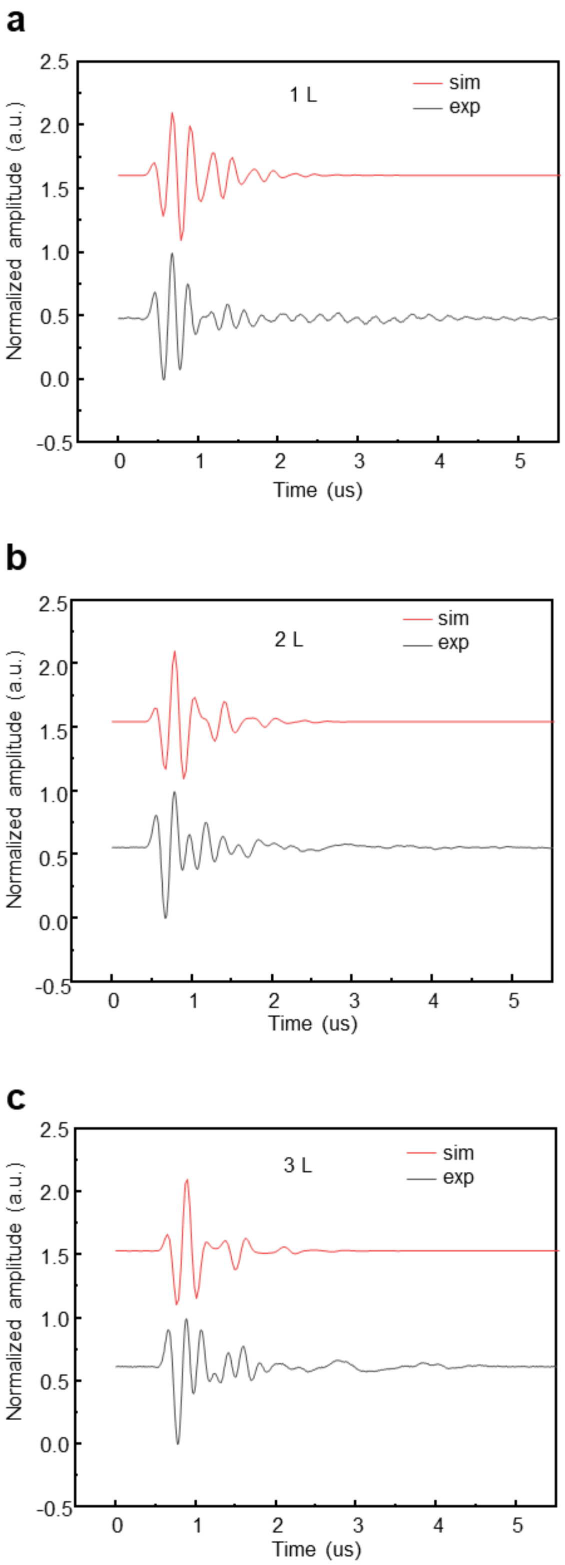

Figure S8

Characterization of matching layers for the CODA ultrasound array. Pulse echo response for a) 1 layer (1 L) b) 2 layers (2 L) and c) 3 layers (3 L) matching. Red color represents PiezoCAD simulation and black represents experimental results.

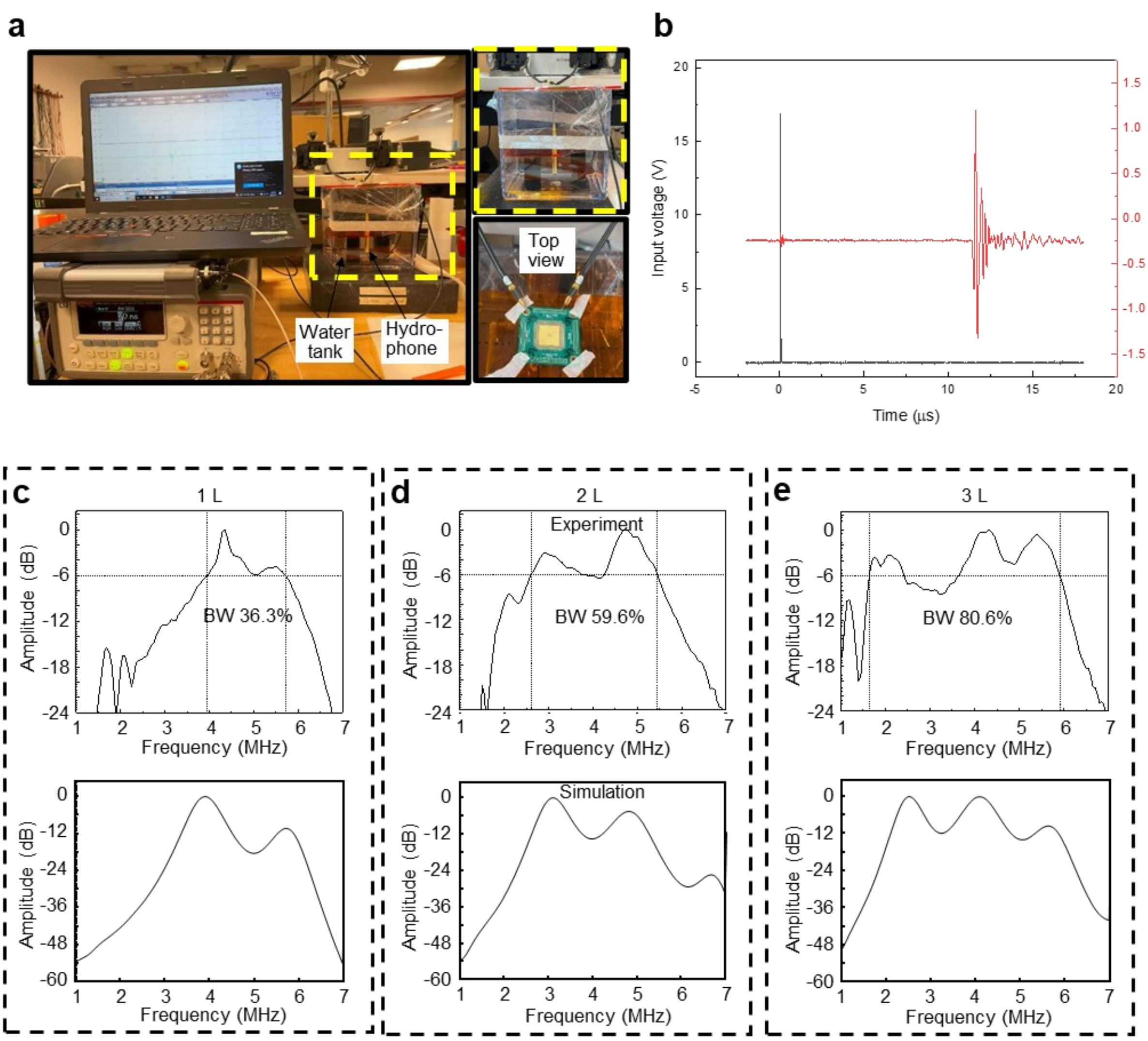

Figure S9

Pulse-echo test to characterize the bandwidth for different matching layers. a) Photo of the experimental setup b) Impulse response when a sinusoidal 1-cycle pulse was applied to an element and output was measured using a needle hydrophone. The black and red lines represent the excitation and hydrophone output signals, respectively. c, d, e) Effect of matching layer on the bandwidth of the ultrasound array, (c) 1 layer (1 L), (d) 2 layers (2 L), and (e) 3 layers (3 L) matching. The bandwidth increased from 36.3% with one layer to 59.6% with two layers and 80.6% with three layers. The bandwidth was calculated using the −6 dB fractional bandwidth criterion with respect to the global peak amplitude of the pulse-echo spectrum. For multilayer matching configurations, multiple local maxima appear due to coupled resonances and interference effects between layers. While valleys between peaks are present, all frequency components within the −6 dB envelope contribute to the effective bandwidth. The 3-layer matching configuration was designed to maximize broadband

response rather than spectral flatness; alternative layer thicknesses could yield smoother frequency responses with reduced bandwidth.

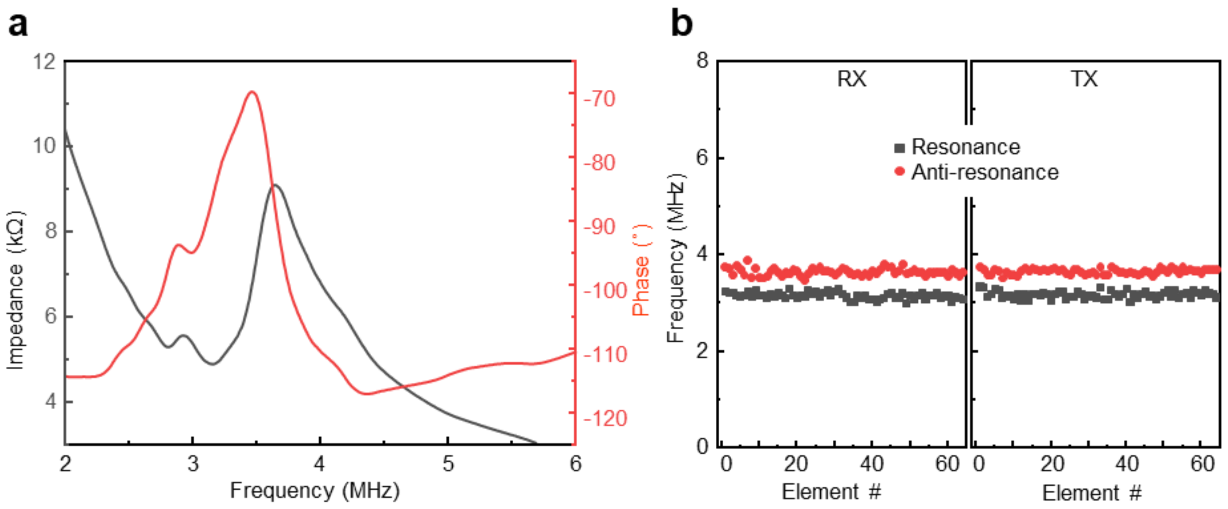

Figure S10

The piezoelectric performance of 2D CODA array. a) Impedance (black) and phase angle (red) spectra of a single element of the CODA array, which utilizes PMN-PT as the piezoelectric material. b) Resonance (black) and anti-resonance (red) frequencies of the 64 transmitter and 64 receiver elements of the CODA array. The element's resonance behavior is highly uniform, which is important for achieving good focusing performance.

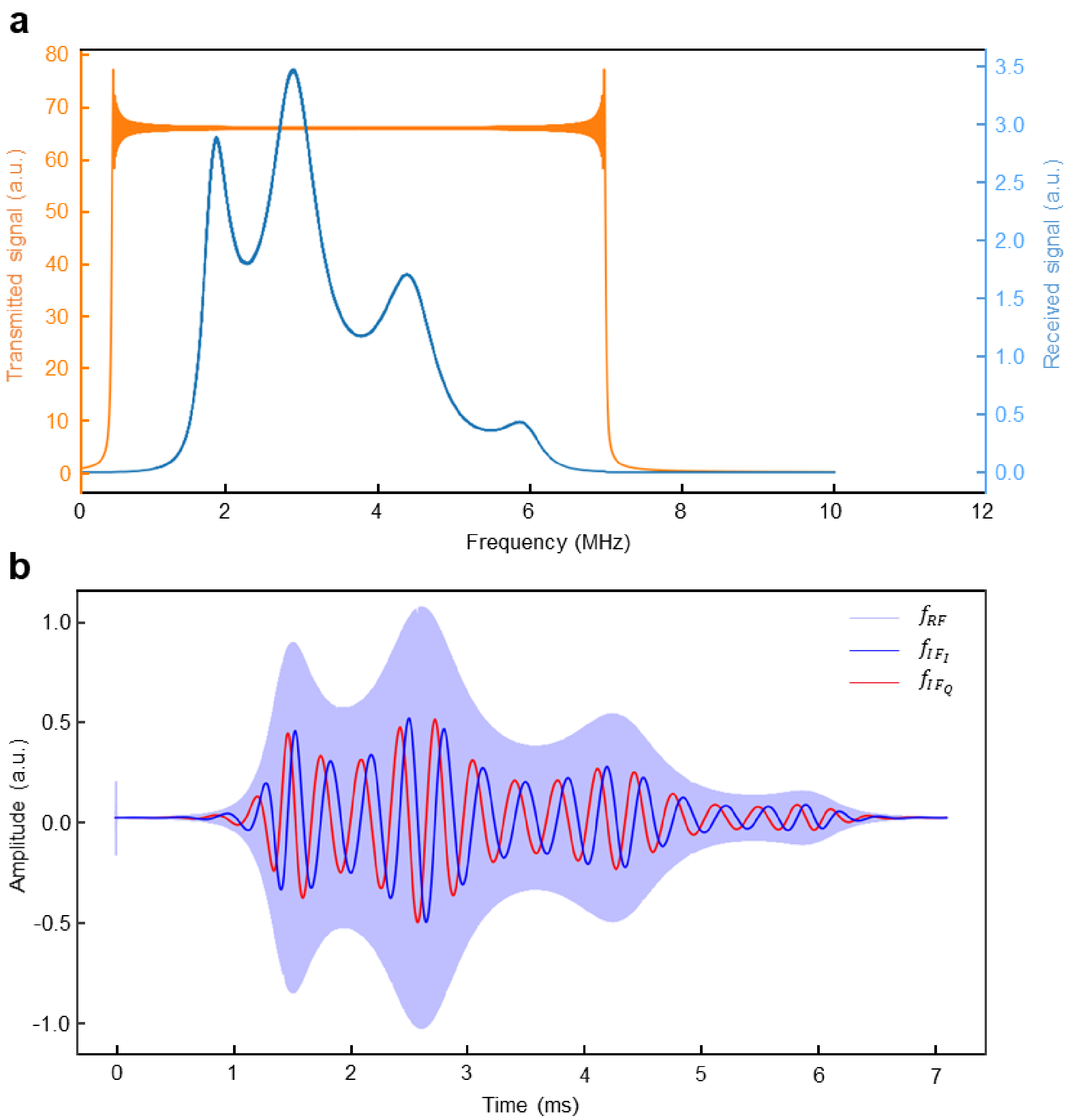

Figure S11

Simulated waveforms in cDAQ System, for a close reflector. a) Transmitted (orange) and received (blue) signals in the frequency domain. The drive signal $f_{RF}(t)$ (orange) is a constant power chirp, shown here sweeping 0.5-7.0 MHz in approximately 7 ms. The received signal $f_{RF}(t - \Delta t)$ (blue) is essentially the pulse-echo frequency response of the piezoelectric transducer. b) Signals shown in the time domain. The received signal $f_{RF}(t - \Delta t)$ (light blue) appears as a solid envelope due to the high frequency. The demodulated and low pass filtered signals f_{IF_I} (red) and f_{IF_Q} (blue) contain the same information, but at a much lower frequency.

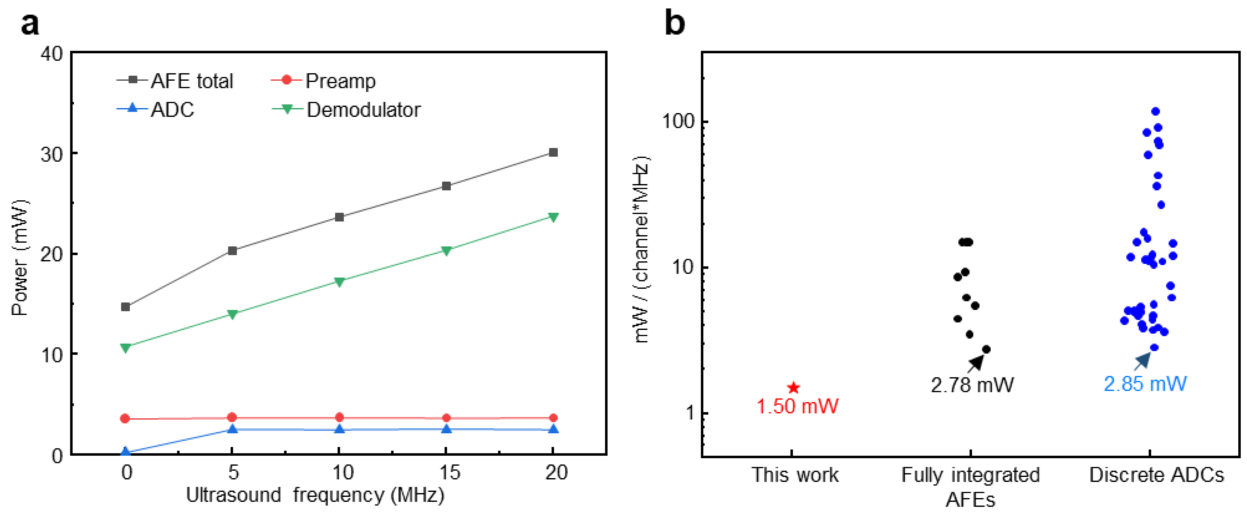

Figure S12

Comparison of per-channel power consumption. a) The power consumption of the cDAQ receive signal chain has a static component due to the amplifiers, plus a dynamic component due to the passive demodulator switches. b) Comparing the power consumption in normalized units of mW/(channel·MHz), our system consumes lower power (1.50) when compared to a sampling of 50 commercially available discrete ADCs (2.85) and fully integrated ultrasound analog front ends (AFEs) (2.78).

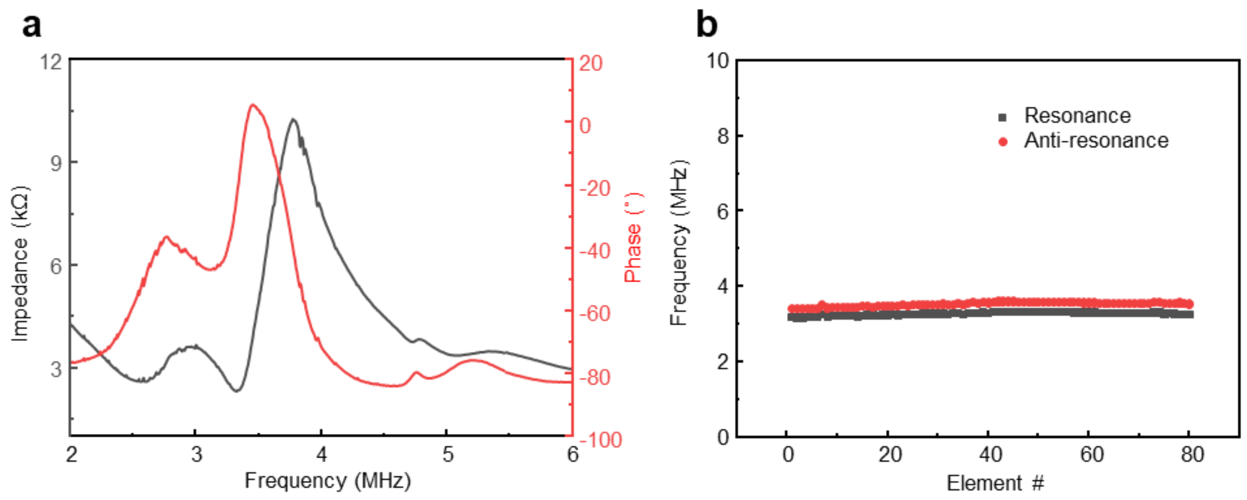

Figure S13

The piezoelectric performance of the 1D array. a) Impedance (black) and phase angle (red) spectra of a single element of 1D array, which utilizes PZT as the piezoelectric material. b) Resonance (black) and anti-resonance (red) frequencies of the 64 elements of the 1D array.

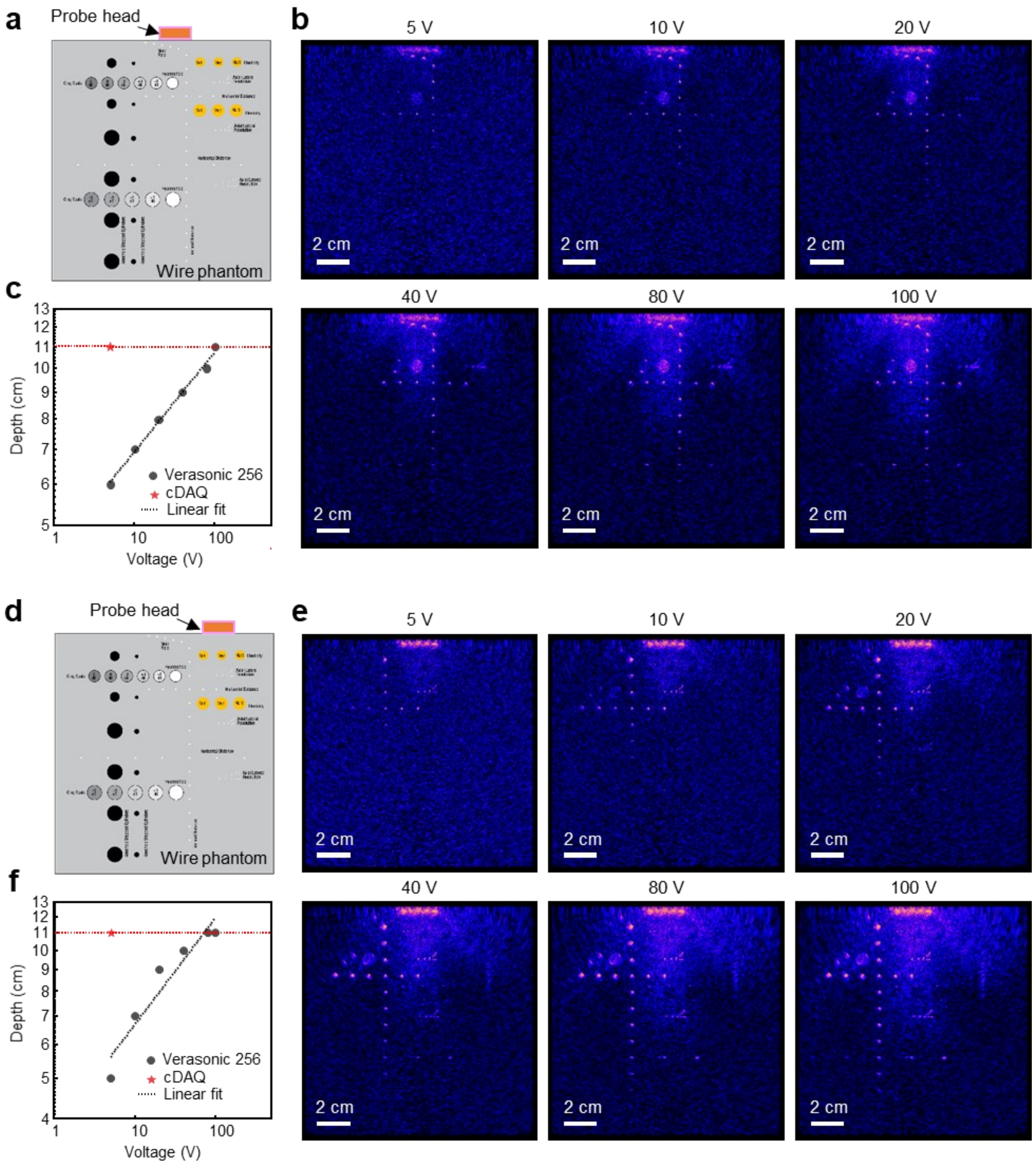

Figure S14

Imaging on wire phantom showing depth as a function of voltage. Images were acquired using Verasonics Vantage 256 system connected to a 1D array. a, b, c) Schematics and corresponding images acquired with the probe positioned above the near-field targets in wire phantom, where (a) shows the schematic of the setup, (b) shows the corresponding ultrasound images, and (c) shows depth versus voltage comparison for Vantage 256 system and our cDAQ system. The cDAQ was operated at 5 V. d, e, f) Schematics and corresponding images acquired with the probe positioned above the resolution targets in wire phantom, where (d)

shows the schematic of the setup, (e) shows the corresponding ultrasound images, and (f) shows depth versus voltage comparison for Vantage 256 system and our cDAQ system. Here, in (c, f) the black line represents the linear fit line for the voltage required to achieve different depths of imaging for the Vantage 256 system and the red line represents depths acquired by the cDAQ at 5 V. The intersection of red and black lines estimates the equivalent voltage required by the Vantage 256 system to achieve the same imaging depth (11 cm).

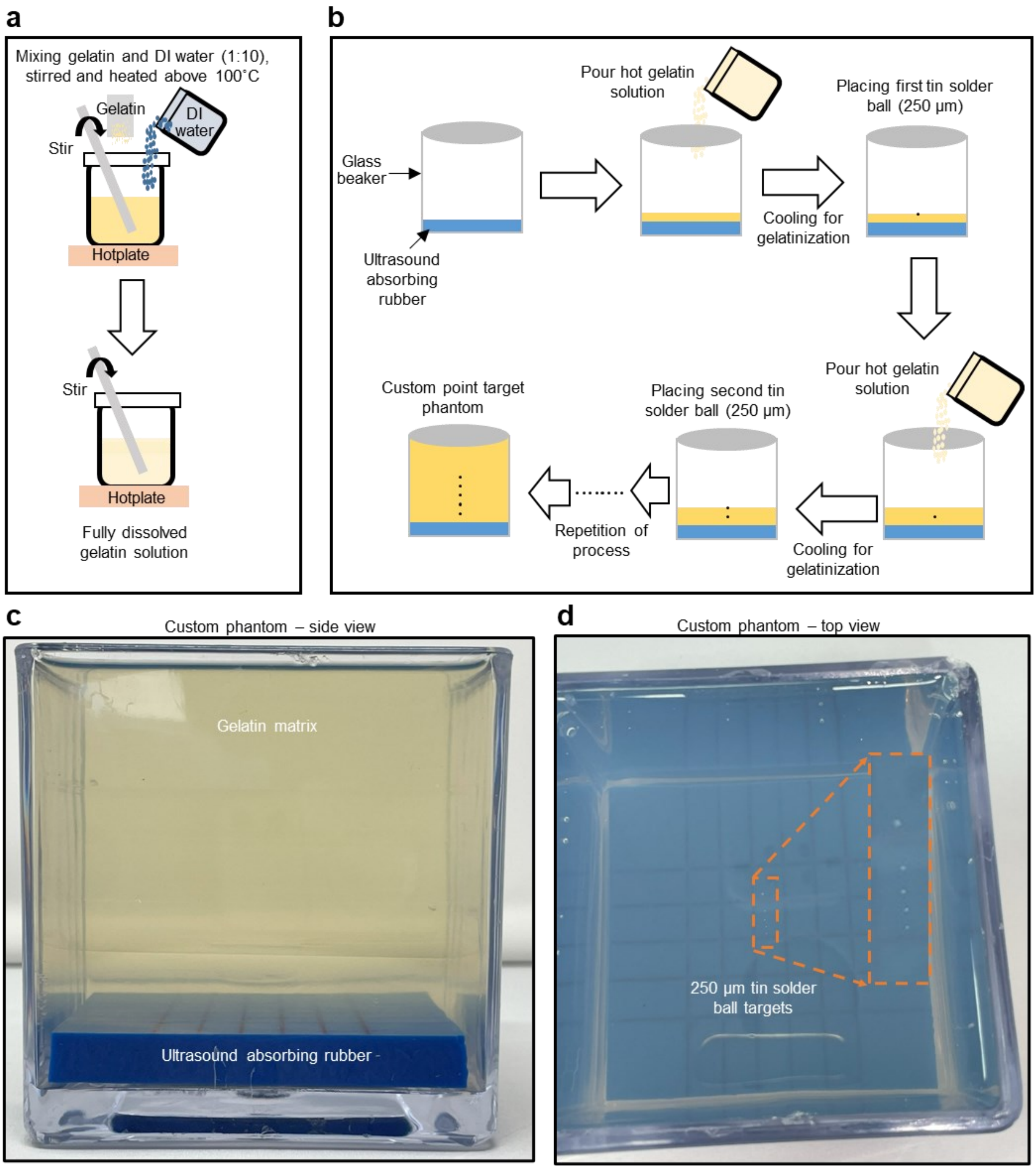

Figure S15

Fabrication of gelatin phantom. a) Preparation of gelatin solution. b) Preparation of gelatin layers and placing tin solder balls layer by layer to make the final phantom with several solder balls aligned vertically. c) Photograph of gelatin phantom (side view). d) Photograph of gelatin phantom (top view).

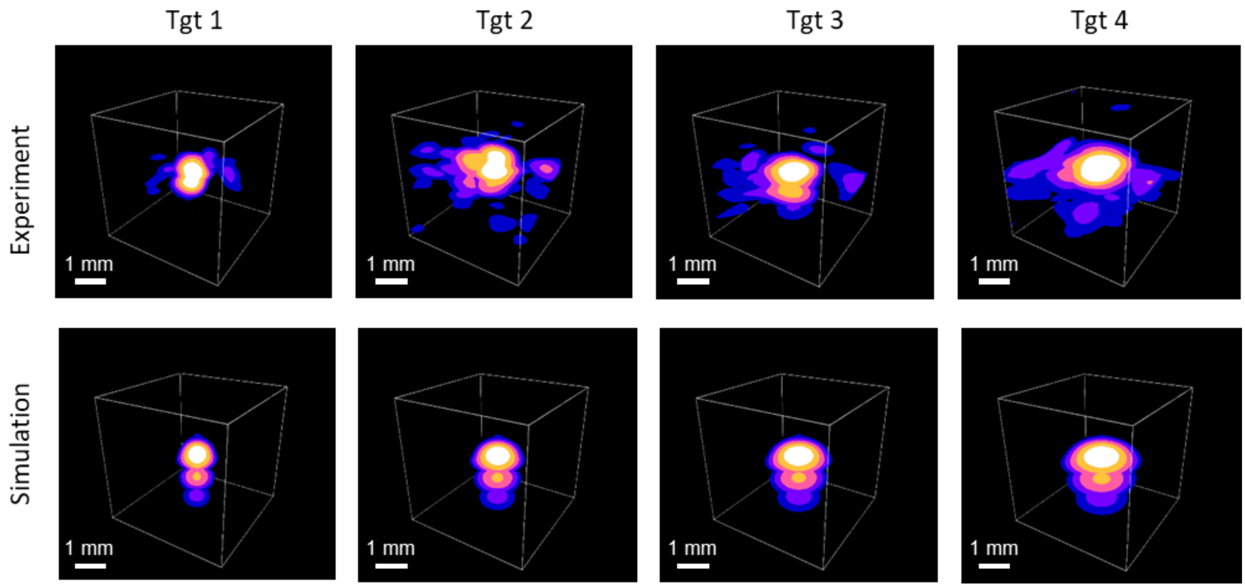

Figure S16

In vitro imaging of 250 µm spherical targets embedded in a custom gelatin phantom to measure the volumetric point spread function (PSF), with experimental results on the top and simulations on the bottom.

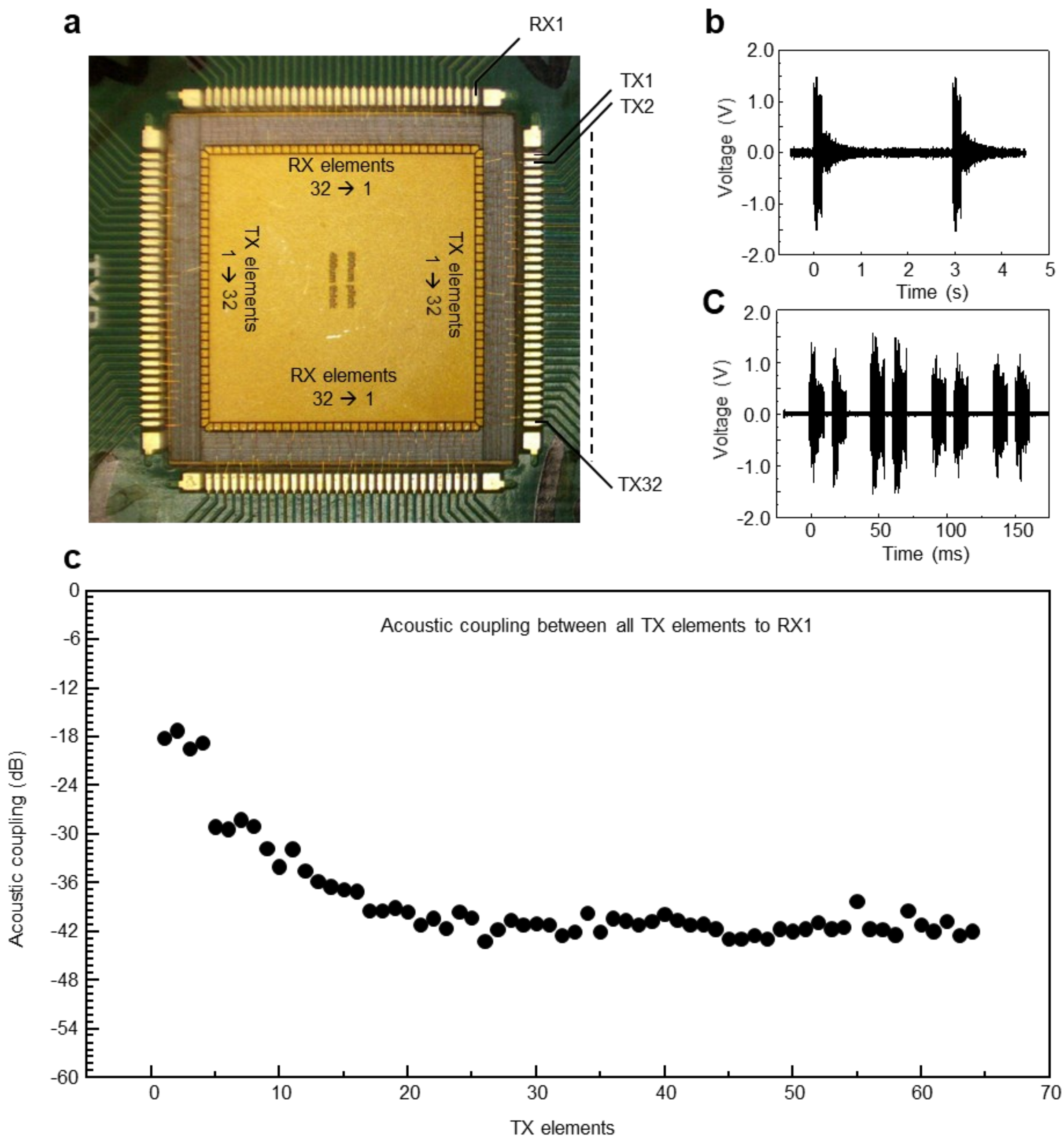

Figure S17

Crosstalk measurement. a) Each transmitter (TX1 - TX64) was driven one at a time and the signal was measured from the low noise amplifier (LNA) output for RX1. b) Received signal when each transmitter was driven with a chirp signal at 18 V c) Zoomed image showing the signals received for TX1 - TX4. d) The measured couplings between TX1 - TX64 and RX1. The coupling appears to taper off due to the noise floor at approximately -40 dB.

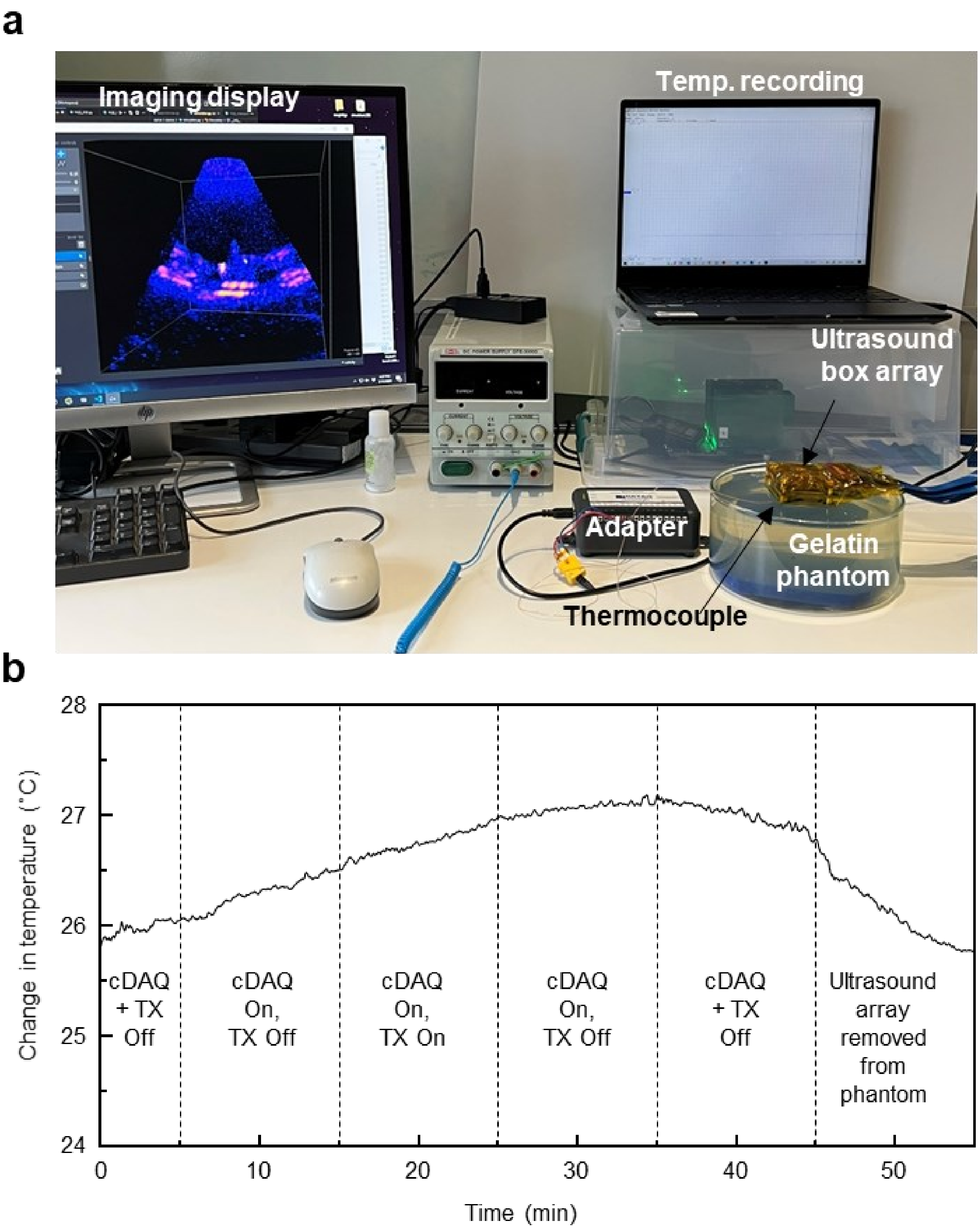

Figure S18

Temperature rise characterization. a) Experimental setup to measure the temperature change using a thermocouple in a gelatin phantom. An 18 V chirp signal was applied to measure the temperature rise. b) Change in temperature under different operating conditions: 0-5 min cDAQ + TX Off, 5-15 min cDAQ On but TX Off, 15-25 min both cDAQ and TX On, 25-35 min cDAQ On but TX Off, 35-45 min both cDAQ and TX Off, 45-55 min ultrasound array removed from the phantom. Over the 55 min of the experiment, the temperature rises only by

1.2 ˚C, which is significantly lower than the FDA limit[28]. In a real-life scenario, during an *in vivo* experiment, the device touching tissue for a few min is sufficient for imaging.

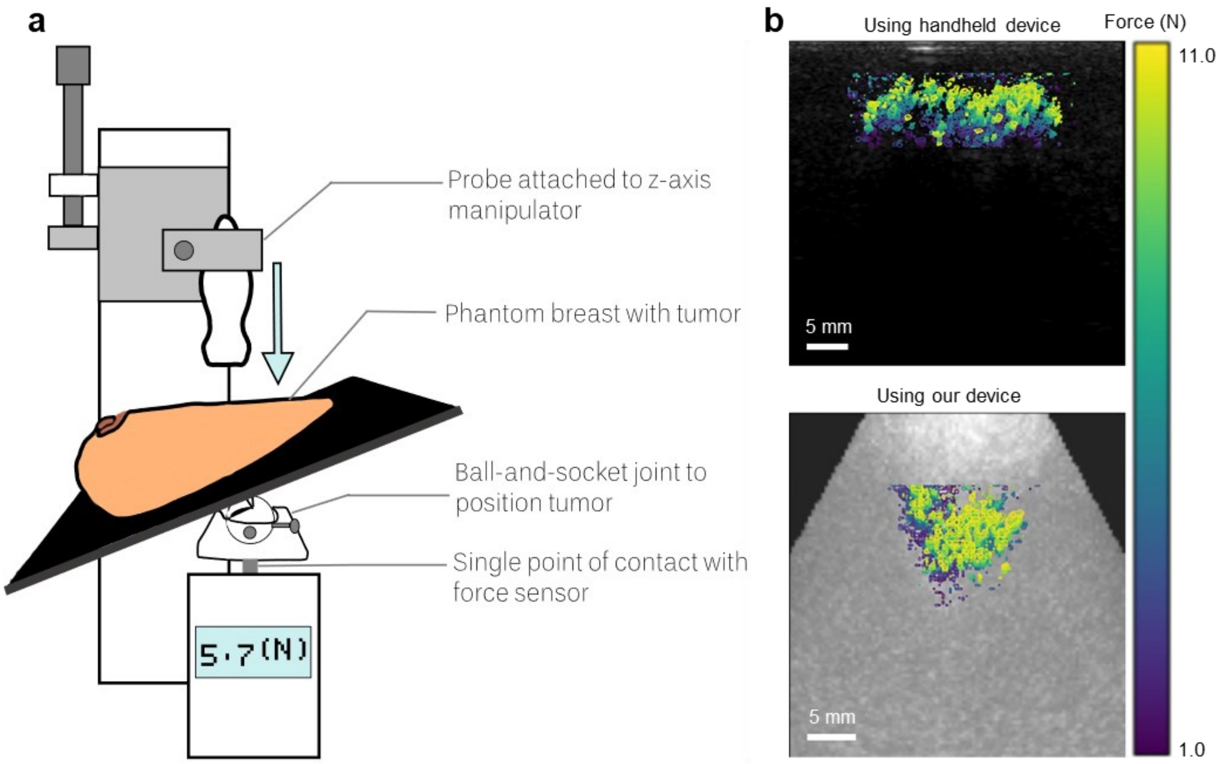

Figure S19

a) Experimental setup used to measure and control probe contact force during imaging. A breast-mimicking phantom with an embedded tumor was mounted on a ball-and-socket joint for adjustable positioning. The ultrasound probe was attached to a z-axis manipulator to ensure repeatable contact, with applied force measured through a single-point load sensor. b) Tumor region from 2D images acquired with a handheld clinical probe. The background corresponds to a single baseline image, while tumor regions imaged at different contact forces were accumulated and overlaid for visualization (top) and corresponding 2D projections of a 3D images acquired using the chirped data acquisition (cDAQ) system and the CODA box-array probe (bottom).

Supplementary video descriptions

Video S1

A real-time 3D ultrasound video of the spiral springs shown in Fig. 1h, captured using the CODA array and cDAQ system.

Video S2

A video captured using the CODA array and cDAQ system, scanning across the tissue-mimicking wire phantom (CIRS, model 040GSE). The video was beamformed in a 2D slice for comparison with standard B-mode imaging.

Video S3

A real-time 3D ultrasound video showing *in vivo* imaging of hyperechoic cysts in breast tissue, captured using the CODA array and cDAQ system.

Table S1

Representative survey of ADCs with 80 MS/s rates, and fully integrated ultrasound analog front end chips. All data was pulled from device datasheets. Note that the power in Column D is specified at the frequency in Column E.

**Name**	**Description**	**ADC Bits**	**Power/channel @ Freq (mW)**	**Freq (MSPS)**	**mW / (channel*MSPS)**
AD6645ASV-80	ADC	14	1500	80	18.75
AD9246BCPZ-80	ADC	14	248	80	3.1
AD9251BCPZ-80	ADC	14	73	80	0.9125
AD9253BCPZ-80	ADC	14	81.5	80	1.01875
AD9255BCPZ-80	ADC	14	239	80	2.9875
AD9265BCPZ-80	ADC	16	241	80	3.0125
AD9266BCPZ-80	ADC	16	113	80	1.4125
AD9269BCPZ-80	ADC	16	100	80	1.25
AD9444BSVZ-80	ADC	14	1200	80	15
AD9446BSVZ-80	ADC	16	2400	80	30
AD9460BSVZ-80	ADC	16	1700	80	21.25
AD9640ABCPZ-80	ADC	14	226	80	2.825
AD9641BCPZ-80	ADC	14	238	80	2.975
AD9644CCPZ-80	ADC	14	211.5	80	2.64375
AD9645BCPZ-80	ADC	14	122	125	0.976
AD9649BCPZ-80	ADC	14	87	80	1.0875
ADC14C080CISQE/NOPB	ADC	14	300	80	3.75
ADC32J43IRGZT	ADC	14	150	80	1.875
ADC34J43IRGZT	ADC	14	203	160	1.26875
ADS5294IPFP	ADC	14	77	80	0.9625
ADS5423IPGP	ADC	14	1850	80	23.125
ADS5542IPAP	ADC	14	545	80	6.8125
ADS5562IRGZT	ADC	16	865	80	10.8125
ADS6143IRHBT	ADC	14	318	80	3.975
ADS6243IRGZT	ADC	14	350	80	4.375

ADS62P43IRGCT	ADC	14	297	80	3.7125
ADS6443IRGCT	ADC	14	75	80	0.9375
LTC1748CFW#PBF	ADC	14	1400	80	17.5
LTC2143CUP-14#PBF	ADC	14	57	80	0.7125
LTC2163CUK#PBF	ADC	16	108	80	1.35
LTC2173IUKG-14#PBF	ADC	14	94	80	1.175
LTC2183CUP#PBF	ADC	16	100	80	1.25
LTC2193IUKG#PBF	ADC	16	125	80	1.5625
LTC2206CUK#PBF	ADC	16	725	80	9.0625
LTC2249CUH#PBF	ADC	14	222	80	2.775
LTC2259IUJ-14#PBF	ADC	14	89	80	1.1125
LTC2266IUJ-14#PBF	ADC	14	101	80	1.2625
LTC2299CUP#PBF	ADC	14	222	80	2.775
LTM9009CY-14#PBF	ADC	14	94	80	1.175
MCP37D21-80E/TE	ADC	14	229	80	2.8625
AFE5816	AFE - full	14	90	65	1.384615385
AFE5808	AFE - full	14	153	65	2.353846154
AD9675	AFE - full	14	150	40	3.75
AD9671	AFE - full	14	150	40	3.75
AD9271	AFE - full	12	150	40	3.75
AFE58JD48	AFE - full	16	140	125	1.12
AFE5828/58JD28	AFE - full	14	102	65	1.569230769
AFE5818/58JD18	AFE - full	14	140	65	2.153846154
AFE5832LP / 58JD32LP	AFE - full	12	27.8	40	0.695
AFE5832 / 58JD32	AFE - full	12	35	40	0.875
